# Vasopressin and oxytocin excite BNST neurons via oxytocin receptors, which reduce anxious arousal

**DOI:** 10.1016/j.celrep.2025.115768

**Published:** 2025-06-03

**Authors:** Walter Francesconi, Valentina Olivera-Pasilio, Fulvia Berton, Susan L. Olson, Rachel Chudoba, Lorena M. Monroy, Quirin Krabichler, Valery Grinevich, Joanna Dabrowska

**Affiliations:** 1 Center for Neurobiology of Stress Resilience and Psychiatric Disorders, Discipline of Cellular and Molecular Pharmacology, The Chicago Medical School, Rosalind Franklin University of Medicine and Science, 3333 Green Bay Road, North Chicago, IL 60064, USA; 2 School of Graduate and Postdoctoral Studies, Rosalind Franklin University of Medicine and Science, 3333 Green Bay Road, North Chicago, IL 60064, USA; 3 Center for Psychiatric Neuroscience, Department of Psychiatry and Behavioral Sciences, Northwestern University, Chicago, IL 60611, USA; 4 Neuroscience Program, Lake Forest College, Lake Forest, IL 60045, USA; 5 Department of Neuropeptide Research in Psychiatry, German Center for Mental Health (DZPG), Medical Faculty Mannheim, Heidelberg University, 68159 Mannheim, Germany; 6 These authors contributed equally; 7 Lead contact

## Abstract

Interoceptive signals dynamically interact with the environment to shape appropriate defensive behaviors. Hypothalamic hormones arginine-vasopressin (AVP) and oxytocin (OT) regulate physiological states, including water and electrolyte balance, circadian rhythmicity, and defensive behaviors. Both AVP and OT neurons project to the bed nucleus of stria terminalis (BNST), which expresses OT receptors (OTRs) and vasopressin receptors, and governs fear responses. However, understanding the integrated role of AVP and OT is complicated by their cross-reactivity and their mutual receptor promiscuity. Here, we provide evidence that the effects of neurohypophysial hormones on BNST excitability are driven by cell-type-specific receptor selectivity and input specificity. We show that OTR-expressing BNST neurons, excited by hypothalamic AVP and OT inputs via OTR, play a major role in regulating BNST excitability, overcoming threat avoidance, and reducing threat-elicited anxious arousal. Therefore, OTR-BNST neurons are perfectly suited to drive the dynamic interactions balancing external threat risk and physiological needs.

## In brief

AVP and OT regulate essential physiological functions. Francesconi and Olivera-Pasilio et al., demonstrate that AVP and OT inputs excite the BNST via OTR. The OTR-expressing BNST neurons reduce anxious arousal, helping to balance external-threat risk with internal physiological demands.

## Graphical Abstract

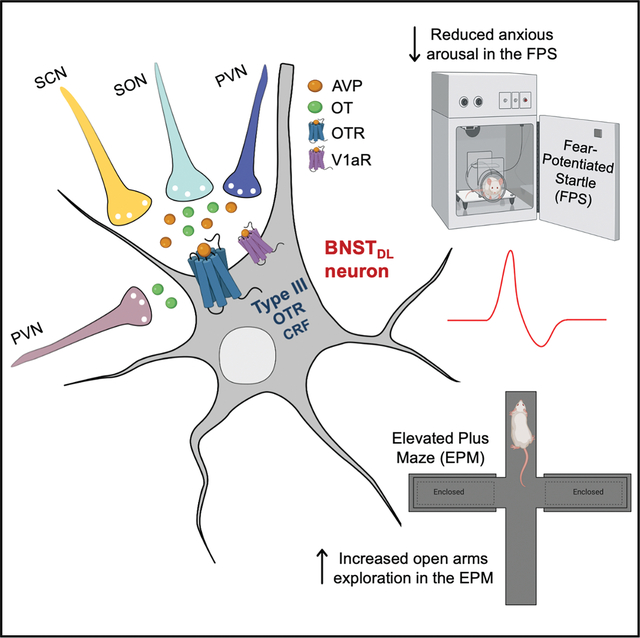

## INTRODUCTION

Interoceptive signals dynamically interact with the environment to shape appropriate defensive behaviors. Rodents avoid open spaces to escape from predators but must overcome this defensive behavior to locate water when driven by thirst. Further, circadian rhythm-associated internal signals drive animals to follow wake-sleep cycles despite predator threats. Numerous physiological functions, including water and electrolyte balance and circadian rhythmicity, are supported by the hormone and neuromodulator arginine-vasopressin (AVP),^1,2^ making AVP a prime candidate for modulating the interaction balancing external risks and internal needs.

AVP signals via three central receptors: vasopressin receptors (V1aR, V1bR) and the oxytocin receptor (OTR), all implicated in defensive behaviors, including fear, avoidance, and anxiety; for review see Serradeil-Le Gal et al.,^3^ Caldwell et al.,^4^ Veenema,^5^ Jurek and Neumann,^6^ Janeček and Dabrowska,^7^ and Olivera-Pasilio and Dabrowska^8^ (of note, V2R is found in the kidneys^9^). These receptors are differentially expressed, with V1bR primarily in the pituitary and select brain regions,^10,11^ whereas V1aRs and OTRs are present throughout the central nervous system^12–14^. Notably, OTR and V1aR seemingly have opposite effects on exploration and fear in rodents,^15,16^ suggesting receptor selectivity as a key mechanism in AVP modulation of defensive behaviors. The extended amygdala, including the central amygdala (CeA) and medial amygdala (MeA), as well as the bed nucleus of stria terminalis (BNST), has a high density of both OT- and AVP-binding sites,^17^ but the specific contributions of each receptor to behavior remain unclear. For example, some regions, like the posterior BNST, show sparse V1aR despite dense AVP fibers and OTR binding.^18^ In addition, commonly used V1aR antagonists also bind OTRs,^19,20^ potentially masking OTR’s role in AVP signaling. Given the abundance of OTRs and V1Rs in the hypothalamus and the extended amygdala, AVP’s effects via OTR might be vastly underestimated.

Beside receptor selectivity, the diverse AVP effects may also depend on input specificity. AVP is produced in hypothalamic neurons—including the paraventricular nucleus (PVN), supraoptic nucleus (SON), and suprachiasmatic nucleus (SCN)^2,21^and the extended amygdala—including the MeA and posterior BNST.^22^ Since AVP and OT largely exist in separate neurons within the hypothalamus,^23^ their inputs, prompted by specific physiological cues, might orchestrate the balance between physiological states and defensive behaviors via downstream neurocircuitries. Given the hypothalamus’ role in assessing internal physiological states and the extended amygdala’s role in gauging external threats, AVP and OT likely flexibly regulate defensive behaviors based on the interplay between physiological needs and external threats.

The BNST is critical for fear processing and vigilant threat monitoring^24–27^ and is one of the few brain regions with high expression of OT and AVP binding sites and fibers,^17,28^ as well as the OTR and V1aR.^29–31^ It receives inputs from hypothalamic OT (primarily PVN) and AVP neurons,^22,29,32^ although the origins of AVP projections to the BNST remain elusive. Primarily a GABAergic and peptidergic structure,^33,34^ dorsolateral BNST (BNST_DL_) contains three major neuron types (types I–III) defined by their intrinsic membrane properties and firing patterns.^35,36^ OT is released in the BNST_DL_ in response to fear stimuli,^37^ where it has neuron-type-specific effects on excitability and synaptic transmission.^38^ However, how the AVP and OT interact via V1R and/or OTR to regulate neuronal excitability and fear processing in the BNST_DL_ remains unclear.

In this study, we used slice electrophysiology, neuronal tract tracing, and peptide optogenetics in naive and AVP-Cre transgenic male rats to show that AVP neurons from the SCN, SON, and PVN project to the BNST_DL_. Both exogenous and evoked AVP excited types I and III BNST_DL_ neurons, which required OTR transmission. Using OTR-Cre transgenic rats,^39,40^ we confirmed that fluorescent OTR-BNST_DL_ neurons, which match the properties of types I and III cells, were excited by AVP. Single-cell PCR previously showed that type III BNST_DL_ neurons express both OTR and corticotropin releasing factor (CRF) mRNA.^29^ Using CRF-Cre transgenic rats,^41^ we demonstrate that AVP and an OTR agonist TGOT directly excites CRF-BNST_DL_ neurons. Finally, chemogenetic silencing of OTR-BNST_DL_ neurons increased anxious arousal in fear-potentiated startle (FPS) and reduced exploration in the elevated-plus maze (EPM), supporting an overall anxiolytic-like function of OTR-BNST_DL_ neurons. These findings show that AVP and OT effects on BNST_DL_ excitability depend on cell-type-specific receptor expression and hypothalamic input specificity. Therefore, changes in hypothalamic activity, sensitive to physiological factors such as water/electrolyte balance or light-dark cycle, will shape BNST_DL_ excitability to balance exploratory and defensive behaviors.

## RESULTS

The treatment effects on intrinsic membrane properties of types I–III BNST_DL_ neurons are in Table S1.

### AVP excites type I BNST_DL_ neurons, which requires OTR, but not V1aR or V1bR

**Figure 1.**
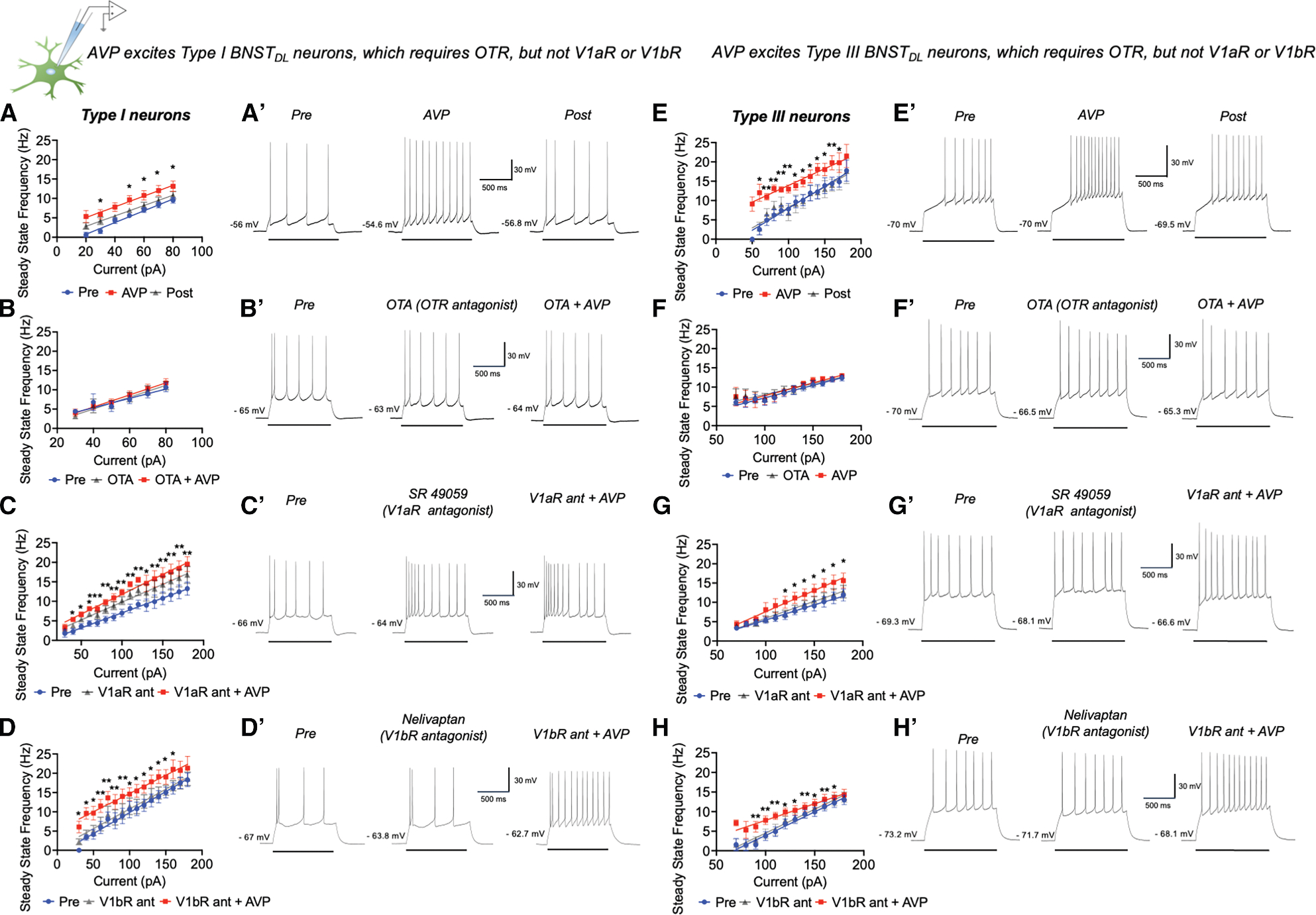
AVP increases intrinsic excitability of type I and III BNST_DL_ neurons, which requires OTR, but not V1aR or V1bR transmission (A) In type I neurons, AVP induced a leftward shift of the I/O relationship, without affecting its slope (*p* = 0.0080, *n* = 9), which recovered during washout. (A′) Traces of type I neuron responses to 40-pA depolarizing current pulse. (B) OTR antagonist, OTA, blocked the excitatory effect of AVP in type I neurons (*p* = 0.0574, *n* = 9). (B′) Type I neuron responses to 60-pA current pulse in the presence of OTA. (C) With V1aR antagonist, SR49059, AVP significantly changed the I/O relationship (*p =* 0.0002, *n* = 9). (C′) Type I neuron responses to 100-pA current pulse in the presence of SR49059. (D) With V1bR antagonist, Nelivaptan, AVP shifted the I/O relationship (*p* < 0.0001, *n* = 10). (D′) Type I neuron responses to 80-pA current pulse during Nelivaptan. (E) In type III neurons, AVP induced a leftward shift of the I/O relationship, without affecting its slope (*p* = 0.0005, *n* = 11), which recovered during washout. (E′) Type III neuron responses to 140-pA depolarizing current pulse. (F) OTA blocked AVP effect on the I/O relationship (*p* = 0.3949, *n* = 9). (F′) Type III neuron response to 100-pA current pulse during OTA. (G) With V1aR antagonist, SR49059, AVP changed the I/O relationship (*p* = 0.0068, *n* = 7). SR49059 alone had no effect on the SSF. (G′) Type III neuron response to 140-pA current pulse during SR49059. (H) AVP shifted the I/O relationship in a presence of V1bR antagonist, Nelivaptan (*p* < 0.0001, *n* = 10). (H′) Type III neuron responses to 110-pA current pulse during Nelivaptan. Electrophysiological data are presented as mean ± standard error of mean (SEM). *p* < 0.05, **p < 0.01, ***p < 0.001 vs. pre.

BNST_DL_ neurons do not fire spontaneously during *in vitro* whole-cell patch-camp electrophysiology.^36,38^ Thus, we measured steady-state-firing frequency (SSF) as a function of current (input/output [I/O] relationship) before (pre), during, and after (washout, post) AVP application. SSF increased incrementally with current, *p* < 0.0001, F(1.316, 10.53) = 63.51, mixed-effects analysis unless stated otherwise, and AVP induced a leftward shift in the I/O relationship, *p* = 0.0080, F(1.531, 12.24) = 8.236, without affecting its slope (no current-treatment interaction) *p* = 0.4596, F (2.120, 12.72) = 0.8421, *n* = 9 neurons from eight rats (Figures 1A and 1A’). Pretreatment with the OTR antagonist OTA, blocked AVP’s effects, showing a significant current, *p* < 0.0001, F(1.341, 10.73) = 59.21, *n* = 9, 8 rats, but no treatment effect, *p* = 0.0574, F(1.466, 11.73) = 3.999, or interaction, *p* = 0.1642, F(1.688, 8.440) = 2.281 (Figures 1B and 1B’). Conversely, in the presence of the V1aR antagonist, SR49059, AVP still shifted the I/O relationship, *p =* 0.0002, F(1.886, 15.09) = 16.29, in addition to a current effect, *p* < 0.0001, F(1.312, 10.49) = 52.74, *n* = 9, 8 rats, with no interaction, *p* = 0.1137, F (1.708, 7.075) = 3.065. Notably, SR49059 showed an excitatory effect on its own vs. pre at 70 pA (*p* = 0.0323), 80 pA (*p* = 0.0221), 90 pA ( *p* = 0.0370), 100 pA ( *p* = 0.0328), 110 pA ( *p* = 0.0328), 120 pA ( *p* = 0.0376), 150 pA ( *p* = 0.0397), 160 pA ( *p* = 0.0432), 170 pA ( *p* = 0.0493), and 180 pA ( *p* = 0.0374) (Figures 1C and 1C’). Pretreatment with V1bR antagonist, Nelivaptan did not block AVP’s effects, *p* < 0.0001, F(1.651, 14.86) = 42.15, while current effect remained, *p* < 0.0001, F (2.294, 20.65) = 117.9, *n* = 10, 8 rats, with no interaction, *p* = 0.4980, F(1.027, 5.475) = 0.5382 (Figures 1D and 1D’).

These results indicate that AVP excites type I neurons through OTR transmission, while V1aR transmission contributes to tonic inhibition in a subset of type I neurons.

### AVP does not affect intrinsic excitability of type II BNST_DL_ neurons

Despite a significant incremental current effect on spike frequency in type II neurons, *p* < 0.0001, F(1.256, 8.789) = 79.04, *n* = 8, 7 rats, AVP did not affect SSF, *p* = 0.1749, F(1.173, 8.210) = 2.211, and there was no interaction, *p* = 0.3589, F (1.414, 8.200) = 1.074, *n* = 8 (not shown).

### AVP excites type III BNST_DL_ neurons, which requires OTR, but not V1aR or V1bR

#### The effect of AVP on the intrinsic excitability of type III BNST_DL_ neurons

**Figure 2.**
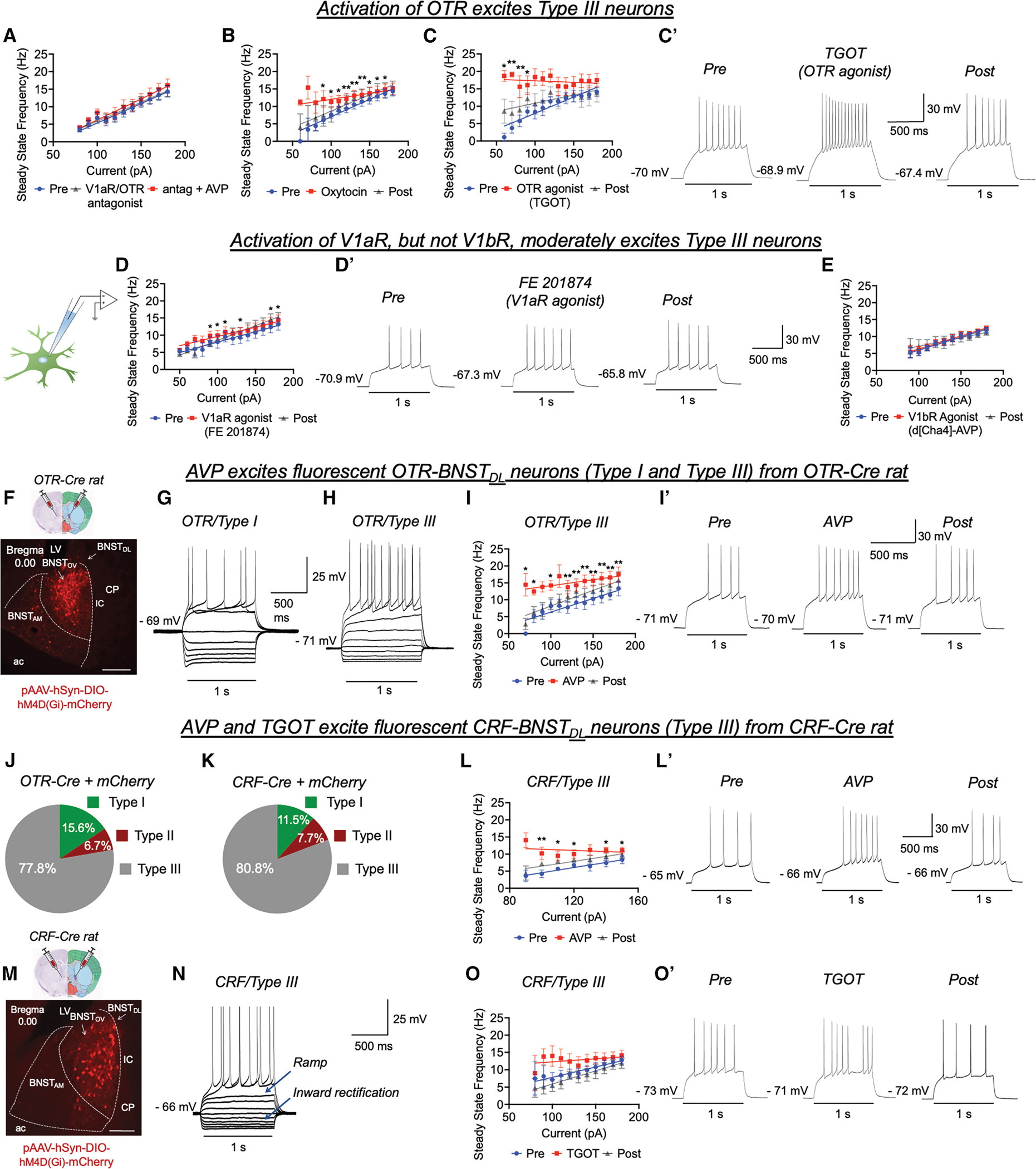
Activation of OTR robustly excites OTR and CRF-expressing type III BNST_DL_ neurons, whereas activation of V1aR, but not V1bR, has a modest excitatory effect (A) In type III neurons, AVP with the V1aR/OTR antagonist did not change the I/O relationship (*p* = 0.2742, *n* = 8). (B) OT induced a leftward shift in the I/O relationship (*p* = 0.0012, *n* = 8). (C) OTR agonist, TGOT, shifted the I/O relationship (*p* = 0.0125, *n* = 8). (C′) Type III neuron responses to a 90-pA pulse during TGOT. (D) V1aR agonist, FE201874, induced an excitatory effect on the I/O relationship (*p =* 0.0071, *n* = 10). (D′) Type III neuron responses to 90-pA pulse during FE201874. (E) V1bR agonist, d[Cha4]-AVP, did not affect the I/O relationship (*p* = 0.3535, *n* = 8). (F) Nissl (left) and anatomical annotations (right) from the Allen-Brain-Atlas.^42^ High somatodendritic OTR-mCherry expression in the BNST_DL_ of OTR-Cre rats injected with AAV-DIO-DREADDs-mCherry. (G and H) Responses of fluorescent-mCherry-OTR (G) type I and (H) type III/OTR neuron to hyperpolarizing and depolarizing current-pulses. (I) In type III/OTR-mCherry neurons, AVP significantly increased SSF (*p* = 0.0003, *n* = 12). (I′) Type III/OTR-mCherry neuron responses to 130-pA pulse during AVP. (J) The majority of OTR-BNST_DL_-mCherry neurons were classified as type III (35/45). (K) The majority of CRF-BNST_DL_-mCherry neurons (21/26) were also type III. (L) In type III/CRF neurons, AVP increased SSF (*p* = 0.0432, *n* = 7). (L′) Type III/CRF neuron responses to 100-pA current pulse during AVP. (M) Nissl (left) and anatomical annotations (right) from the Allen-Brain-Atlas.^42^ High CRF-mCherry expression in the BNST_DL_ after CRF-Cre rats were injected with AAV-DIO-DREADDs-mCherry. (N) Responses of type III/CRF-mCherry neuron to hyperpolarizing and depolarizing currents. (O) In type III/CRF neurons, TGOT significantly increased SSF (*p* = 0.0211, *n* = 7), with trends at 140 pA (*p* = 0.0697) and 150 pA (*p* = 0.0780, vs. pre). (O′) Type III/CRF-mCherry neuron responses to 150-pA pulse during TGOT. Electrophysiological data are presented as mean ± standard error of mean (SEM). *p < 0.05, **p < 0.01, ***p < 0.001 vs. pre. ac, anterior commissure; BNST_AM_, anteromedial BNST; BNST_OV_, oval nucleus of the BNST; CP, caudate putamen; IC, internal capsulae; LV, lateral ventricle.

There was an incremental current effect on SSF, *p* = 0.0003, F (1.031, 10.31) = 29.13, *n* = 11, 10 rats, AVP induced a leftward shift of the I/O relationship, *p* = 0.0005, F(0.9127, 9.127) = 29.40, without affecting its slope, *p=*0.1426, F(1.586, 5.125) = 2.955 (Figures 1E and 1E’). In the presence of OTA, an incremental current effect remained, *p* < 0.0001, F(1.877, 15.01) = 61.34, *n* = 9, 5 rats, but no treatment effect of AVP, *p* = 0.3949, F(1.419, 11.35) = 0.9149, and no interaction, *p* = 0.3986, F(0.6373, 1.880) = 0.8231 (Figures 1F and 1F’). In contrast, with the V1aR antagonist SR49059, there was a current effect, *p* = 0.0003, F(0.9437, 5.662) = 62.36, *n* = 7, 7 rats, and AVP still shifted the I/O relationship, *p* = 0.0184, F(1.166, 6.997) = 8.869, with no interaction, *p* = 0.3410, F(1.224, 4.841) = 1.202. Application of SR49059 alone had no effect on SSF at any injected current (*p* > 0.05) (Figures 1G and 1G’). Pretreatment with V1bR antagonist, Nelivaptan, did not block AVP’s effect on SSF, *p* < 0.0001, F(1.651, 14.86) = 42.15, while current effect remained, *p* < 0.0001, F(2.294, 20.65) = 5117.9, *n* = 11, 10 rats, with no interaction, *p* = 0.4980, F(1.027, 5.475) = 0.5382 (Figures 1H and 1H’). Finally, in the presence of a V1aR/OTR antagonist, SSF increased with current, *p* < 0.0001, F(1.543, 10.80) = 55.46, *n* = 8, 8 rats, but AVP no longer changed the I/O relationship, *p* = 0.2742, F(0.6225, 4.358) = 1.293 (Figure 2A).

Since OTR, but not V1aR or V1bR blockade, abolished AVP’s excitatory effects on type III neurons, these findings indicate that AVP excites type III neurons via OTR transmission.

#### The effect of OTR activation on intrinsic excitability of type III BNST_DL_ neurons

OT induced a leftward shift of the I/O relationship, *p* = 0.0012, F (1.793, 12.55) = 2.79, *n* = 8, 8 rats. There was an incremental current effect on SSF, *p* < 0.0001, F(1.487, 10.41) = 93.01, and a current-treatment interaction, *p* = 0.0172, F(2.584, 10.23) = 5.687 (Figure 2B), suggesting that OT affected the slope of the I/O curve. Similarly, in the presence of OTR agonist, TGOT, there was a current effect on SSF, *p* = 0.0001, F(1.784, 12.49) = 22.26, *n* = 8, 5 rats, and a treatment effect, *p* = 0.0125, F (1.026, 7.184) = 10.82, with a trend in the current-treatment interaction, *p* = 0.0840, F(2.457, 5.323) = 3.975 (Figures 2C and 2C’).

#### The effect of V1aR and V1bR activation on intrinsic excitability of type III BNST_DL_ neurons

When FE201874, a specific V1aR agonist, was tested, there was a current, *p* < 0.0001, F(1.940, 17.46) = 64.79, *n* = 10, 9 rats, and a treatment effect on SSF, *p* = 0.0071, F(1.546, 13.91) = 8.098, with no interaction, *p* = 0.1757, F(2.690, 8.174) = 2.126 (Figures 2D and 2D’). In the presence of V1bR agonist, d [Cha4]-AVP, there was an incremental current effect on spike frequency, *p* < 0.0001, F(1.127, 7.890) = 242.0, *n* = 8, 4 rats, with no treatment effect, *p* = 0.3535, F(0.2997, 2.098) = 0.3751, and no interaction, *p* = 0.2580, F(1.286, 4.572) = 1.755 (Figure 2E).

These results show that OTR activation alone, or OTR/V1aR activation, robustly excites type III neurons, whereas V1aR activation alone has a moderate excitatory effect.

### AVP and TGOT excite type III/CRF and type III/OTR neurons from transgenic rats

#### AVP excites type III/OTR neurons from OTR-Cre rats

Intrinsic excitability of fluorescent OTR neurons was measured as SSF in slices obtained from OTR-Cre rats injected with AAV-DIO-DREADDs-mCherry in the BNST_DL_. First, fluorescent OTR-mCherry neurons were characterized as types I–III neurons based on their electrophysiological properties. We recorded from a total of 45 OTR-mCherry neurons, of which 7 (15.6%) were characterized as type I, 3 (6.7%) as type II, and 35 (77.8%) as type III (Figures 2G, 2H, and 2J). In type III/OTR neurons, mixed-effect analysis revealed a current effect on spike frequency, *p* < 0.0001, F(1.481, 16.29) = 34.58, *n* = 12, 11 rats, and AVP increased SSF, *p* = 0.0003, F(1.282, 14.11) = 20.25, with a current-treatment interaction, *p =* 0.0199, F(2.698, 15.58) = 4.566 (Figures 2I and 2I’).

#### AVP excites type III/CRF neurons from CRF-Cre rats

The majority of type III BNST_DL_ neurons express CRF mRNA.^33,34^ Thus, we tested AVP effects on CRF neurons recorded in slices obtained from CRF-Cre rats injected with AAV-DIO-DREADDs-mCherry in the BNST_DL_. The great majority of recorded fluorescent CRF-mCherry neurons (*n* = 26) were characterized as type III (*n* = 21 [80.8%]); two were classified as type II (7.7%), and three as type I neurons (11.5%) (Figures 2K and 2N). In type III/CRF neurons, there was a significant current effect on spike frequency, *p* = 0.0093, F(1.724, 10.34) = 8.056, *n* = 7, 4 rats, and AVP showed an incremental effect on SSF, *p* = 0.0432, F(1.167, 7.000) = 5.833, and a current-treatment interaction, *p =* 0.0397, F(2.208, 7.177) = 5.102 (Figures 2L and 2L’).

#### TGOT excites type III/CRFneurons from CRF-Cre rats

In type III/CRF neurons, there was a current effect, *p* = 0.0073, F (1.714, 10.28) = 8.779, *n* = 7, 5 rats, and TGOT induced a significant incremental effect on SSF, *p* = 0.0211, F(1.694, 10.16) = 6.115, with no interaction, *p =* 0.1915, F(2.050, 8.611) = 2.013 (Figures 2O and 2O’). These results show that AVP and TGOT excite OTR and CRF neurons of the BNST_DL_.

### The BNST_DL_ contains numerous OTR neurons, which co-express striatal-enriched protein tyrosine phosphatase or protein kinase C delta

**Figure 3.**
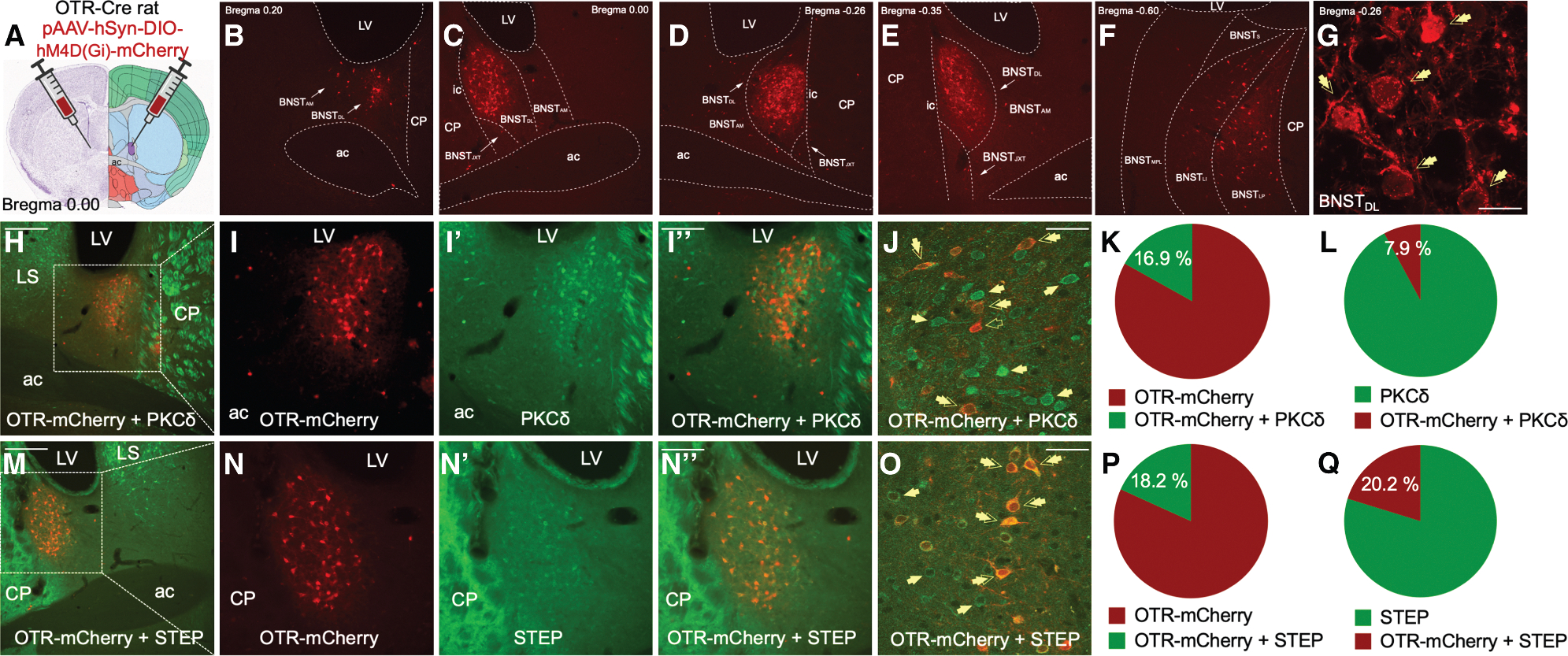
The BNST_DL_ contains numerous OTR-expressing neurons, which co-express STEP or PKCδ (A) Nissl (left) and anatomical annotations (right) from the Allen-Brain-Atlas.^42^ (B–F) OTR-mCherry cell bodies and spiny dendrites are localized in the anterior (B and C), middle (D and E), and posterior (F) BNST, with the majority of OTR-neurons located in the BNST_DL_, especially the BNSTov (C–E) and posterior BNST_DL_ (F), whereas fewer OTR neurons are in anteromedial BNST (BNST_AM_). (G) Confocal images show robust somatodendritic expression of OTR-BNST_DL_-mCherry neurons (original magnification ×60; scale bar, 10 μm). (H–R) Double-immunofluorescence microphotographs show high density of OTR-, PKCδ, and STEP neurons in the BNST_DL_ (original magnification ×10x) (H and M) and co-expression between OTR-PKCδ (original magnification ×20) (I–I″), and OTR-STEP neurons (N–N”). Confocal images (J and O) (original magnification ×60; scale bar, 20 μm) and quantification from multi-tiles-Z-stacks of the entire BNST_DL_ show that OTR-mCherry neurons co-express PKCδ (K and L) or STEP (P and Q).

Numerous OTR-mCherry cell bodies and spiny dendrites were observed in the anterior (bregma 0.20 to −0.00 mm) (Figures 3B and 3C), middle (−0.26 to −0.35 mm) (Figures 3D and 3E), and posterior (−0.60 mm) (Figure 3F) BNST. The majority of the OTR neurons were located in the BNST_DL_, primarily clustered in the oval nucleus (BNSTov, 0.00 to −0.35 mm) (Figures 3C–3E), whereas fewer OTR neurons were found in the anteromedial BNST (Figures 3B–3E). In posterior BNST, OTR neurons were found in lateral-posterior BNST, intermediate-posterior BNST, and to a lesser extent in medial, posterolateral BNST (Figure 3F). Double-immunofluorescence analysis of confocal images showed that 16.91 ± 4.41% of OTR-mCherry-neurons co-expressed protein kinase C delta (PKCδ) (Figure 3K), whereas 7.87 ± 1.08% of all PKCδ neurons co-expressed OTR-mCherry (Figure 3L). Double-immunofluorescence showed that 18.23 ± 2.05% of OTR-mCherry-neurons co-expressed striatal-enriched protein tyrosine phosphatase (STEP) (Figure 3P), whereas 20.22 ± 2.51% of STEP neurons co-expressed OTR-mCherry (Figure 3R). These results suggest that OTR neurons constitute a diverse population of BNST_DL_ neurons.

### OTR-BNST_DL_ neurons are necessary for the extinction of anxious arousal in the FPS

**Figure 4.**
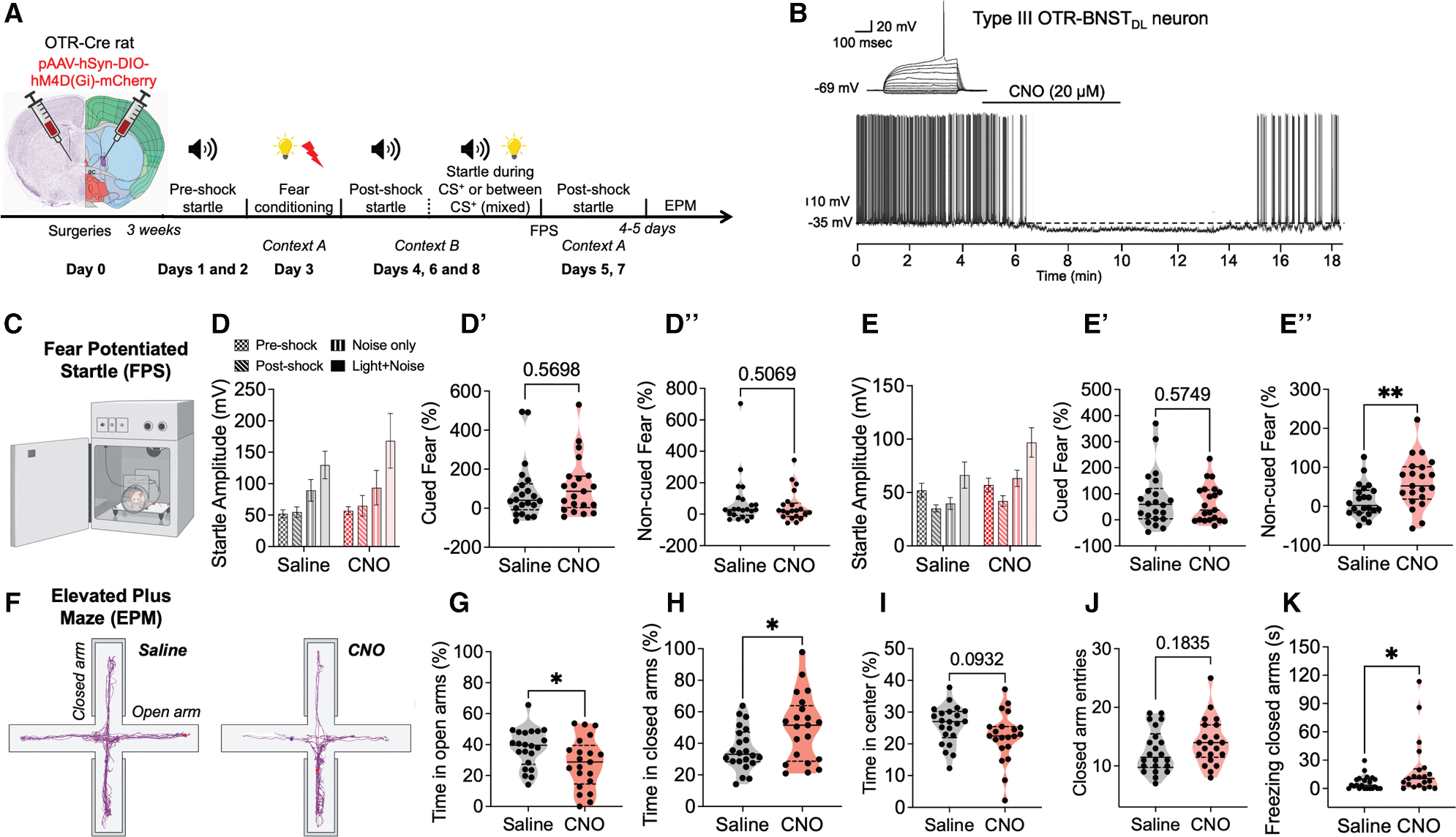
OTR neurons in the BNST_DL_ are necessary for the extinction of anxious arousal in the FPS and open-arms exploration in the EPM (A) Nissl (left) and anatomical annotations (right) from the Allen-Brain-Atlas.^42^ Male OTR-Cre rats injected with AAV-DIO-DREADDs-Gi-mCherry into the BNST_DL_ were treated with CNO or saline before fear-conditioning in the FPS and again, before the EPM. (B) *In vitro* patch-clamp electrophysiology in brain slices from the OTR-Cre rats shows that CNO reduces the firing frequency of OTR-BNST-mCherry neurons. (C) FPS apparatus with Plexiglass enclosure and startle detection. (D–D″) During the first FPS-recall, there was a trial effect of noise-only vs. light and noise (*p* < 0.0001), and post-shock vs. noise-only conditions (*p* = 0.0109), but no treatment effects (D). No significant differences were found in the percentage change of cued fear (D′) or non-cued fear (D″). (E–E″) During the second FPS, there was a trial effect between the noise-only and light and noise conditions (*p* < 0.0001), and a treatment effect (*p* = 0.0477). There was a trial effect between the post-shock and noise-only conditions (*p* = 0.0024), a treatment effect (*p* = 0.0307), and an interaction (*p* = 0.0430) (E). There were no differences in the percentage change of cued fear (E′), but the percentage change of non-cued fear (anxious arousal) was significantly higher in CNO vs. saline controls (*p* = 0.0077) (E″). (F–K) Representative traces of locomotor activity during the EPM (F). Rats treated with CNO spent less time in the open-arms (G), spent more time in the closed-arms (H), and tended to spend less time in the center (I) vs. saline controls. No significant differences were found between groups in the number of closed-arms entries (J). CNO-treated rats spent significantly more time freezing in the closed-arms vs. saline-treated rats (*p* = 0.0344) (K). *p < 0.05, **p < 0.01.

Figure 4A shows the timeline of behavioral experiments and Figure S3 shows FPS components. The inhibitory effect of DREADDs-Gi in OTR-BNST_DL_-mCherry neurons was confirmed with electrophysiology in brain slices containing the BNST from OTR-Cre rats (*n* = 4) injected with an AAV-DIO-DREADDs-Gi-mCherry. Here, application of clozapine-N-oxide (CNO) significantly reduced the firing rate recorded near the threshold for action potentials (Figure 4B).

OTR-Cre rats (*n* = 43) injected with the AAV-DREADDs were used for chemogenetic inhibition of OTR-BNST_DL_ neurons before fear conditioning. Only rats with robust unilateral, high bilateral, or at least moderate bilateral expression of DREADDs-mCherry in OTR-BNST_DL_ neurons were included in the analysis. During fear conditioning, there were no significant differences in foot-shock reactivity between saline- and CNO-injected rats (*p* = 0.1041, saline *n* = 22, CNO *n* = 21; not shown). During the first FPS recall in context B, there was a significant trial effect between the noise-only and light and noise, *p* = 0.0001, F(1, 41) = 18.25, two-way repeated measures (RM) ANOVA, indicating effective cued fear-learning, but no effect of treatment (saline vs. CNO), *p* = 0.5831, F(1, 41) = 0.3060, nor interaction between trial and treatment, *p* = 0.2086, F(1, 41) = 1.632 (Figure 4D). There was a trial effect between the post-shock and noise-only conditions, *p* = 0.0109, F(1, 41) = 7.121, indicating a non-cued fear expression, but no treatment effect, *p* = 0.7613, F(1, 41) = 0.09352, nor interaction, *p* = 0.8035, F(1, 41) = 0.06275, (Figure 4D). During the first contextual fear recall in context A, there was a trial effect between pre-shock and post-shock, *p* = 0.0459, F(1, 41) = 4.239, but no treatment effect, *p* = 0.8054, F(1, 41) = 0.06150, or interaction, *p* = 0.2545, F(1, 41) = 1.336, not shown. During the first FPS recall test, no significant differences were found in the percentage change of cued fear (*p* = 0.5698) (Figure 4D’), non-cued fear (*p* = 0.5069) (Figure 4D”), or contextual fear (*p* = 0.2541; not shown).

During the second FPS recall in context B, there was a trial effect between the noise-only and light and noise conditions, *p* < 0.0001, F(1, 41) = 24.68, and a treatment effect, saline vs. CNO) (*p* = 0.0477, F(1, 41) = 4.168, but no interaction, *p* = 0.5685, F(1, 41) = 0.3305 (Figure 4E). *Post hoc* analysis showed that light and noise startle tended to be higher in the CNO than the saline group (*p* = 0.0768). There was a trial effect between the post-shock and noise-only conditions, *p* = 0.0024, F(1, 41) = 10.52, a treatment effect, *p* = 0.0307, F(1, 41) = 5.009, and a trial-treatment interaction, *p* = 0.0430, F(1, 41) = 4.361. *Post hoc* analysis showed that CNO-treated (*p* = 0.0012) but not saline-treated rats (*p* = 0.6559) (Figure 4E) exhibited a significantly higher ASR in the noise-only than in the post-shock trials. During the second contextual fear test in context A, there was no trial effect between the before the shock and after the shock, *p* = 0.9590, F(1, 41) = 0.002675, no treatment effect, *p* = 0.7974, F(1, 41) = 0.06678, and no interaction, *p* = 0.5209, F(1, 41) = 0.4193, not shown. There were no differences in the percentage change of cued fear (*p* = 0.5749) (Figure 4E’) or contextual fear (*p* = 0.6199, not shown), but there was a significantly higher percentage change of non-cued fear in CNO-treated than in saline-treated rats (*p* = 0.0077) (Figure 4E”).

During the third FPS recall in context B, there was no longer a trial effect between the noise-only and light and noise conditions, *p* = 0.9938, F(1, 41) = 6.080e-005, and no treatment effect, *p* = 0.3467, F(1, 41) = 0.9063. There was still a trial effect between the post-shock and noise-only conditions, *p* = 0.0378, F(1, 41) = 4.605, but no treatment effect, *p* = 0.2787, F(1,41) = 1.205. There were no significant differences in the percentage change of cued fear (*p* = 0.3693) or non-cued fear (*p* = 0.9878, not shown).

These results show that silencing OTR-BNST_DL_ neurons delays the extinction of non-cued fear in the FPS, indicating that OTR-BNST_DL_ neurons are necessary for the extinction of anxious arousal.^43,44^

### OTR-BNST_DL_ neurons are necessary for open-arms exploration in the EPM

After the FPS, the same OTR-Cre rats (*n* = 43) were used to examine the effect of chemogenetic inhibition of OTR-BNST_DL_ neurons on EPM behavior. Examples of the locomotor activity of saline- vs. CNO-treated rat are shown in Figure 4F. No significant differences between saline and CNO-treated rats were found in the number of entries to open arms (*p* = 0.3382, unpaired *t* test, not shown), closed arms (*p* = 0.1835, Figure 4J), or EPM center (*p* = 0.8387, not shown), suggesting that silencing OTR-BNST_DL_ neurons does not affect locomotor activity. Time spent in each compartment was calculated as the percentage of total time in the EPM. CNO-treated rats spent significantly less time in the open arms (*p* = 0.0307) (Figure 4G) and significantly more time in the closed arms (*p* = 0.0168) (Figure 4H) and tended to spend less time at the center (*p* = 0.0932) (Figure 4I) than saline controls. No significant differences were found in time freezing in open arms (*p* = 0.3066, not shown), but CNO-treated rats spent significantly more time freezing in closed arms (*p* = 0.0344) (Figure 4K) than saline-treated rats.

### Hypothalamic AVP neurons from the SON, SCN, and the PVN project to the BNST_DL_

**Figure 5.**
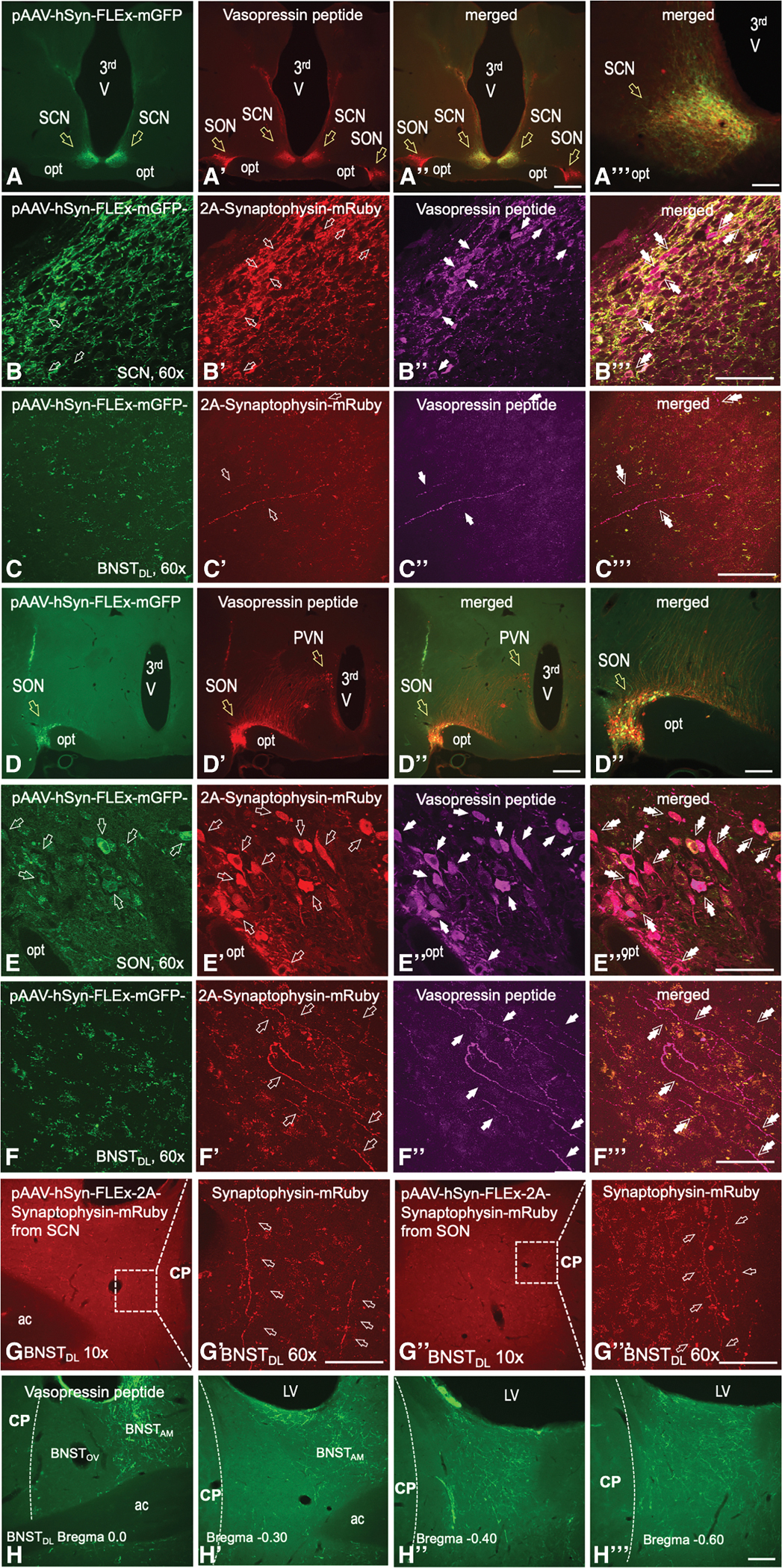
Suprachiasmatic and supraoptic nuclei of the hypothalamus send AVP-peptidergic fibers to the BNST_DL_ AVP-Cre transgenic rats were bilaterally injected with pAAV-hSyn-FLEx-mGFP-2A-synaptophysin-mRuby in the SCN or SON and AVP peptide was visualized with Alexa-Flour 647. (A–A‴) Co-expression of GFP and AVP-peptide (red) in the SCN, but not SON at an original magnification of ×4 (A″; scale bar, 100 μm) and original magnification of ×20 (A‴; scale bar, 25 μm). (B–B‴) Confocal images (original magnification ×60; scale bar, 10 μm) show the co-expression of GFP (B), 2A-synaptophysin-mRuby (red, open arrows, B′), and AVP-peptide (magenta, closed arrows, B″) in SCN neurons (double arrows, B‴). (C–C‴) Confocal images (original magnification ×60) show 2A-Synaptophysin-mRuby (red, open arrows, C′) and AVP peptide (magenta, closed arrows, C″) co-localization in BNST_DL_ fibers originating from the SCN (10 μm, double arrows, C‴). (D–D‴) Co-expression of GFP and AVP-peptide (red) in the SON, but not SCN at an original magnification of ×4 (D″; scale bar, 100 μm) and original magnification of ×20 (D‴; scale bar, 25 μm). (E–E‴) Confocal images (original magnification ×60; scale bar, 10 μm) show co-expression of GFP (E), 2A-synaptophysin-mRuby (red, open arrows, E′), and AVP-peptide (magenta, closed arrows, E″) in SON neurons (double arrows, E‴). (F–F‴) Confocal images (original magnification ×60; scale bar, 10 μm) show co-expression of 2A-synaptophysin-mRuby (red, open arrows, F′) and AVP peptide (magenta, closed arrows, F″) in BNST_DL_ fibers originating from the SON (double arrows, F‴). (G–G‴) Confocal images of mRuby signal alone show BNST_DL_ synaptophysin-mRuby fibers, originating from the SCN (G–G″), or SON (G″ and G‴). (H–H‴). Immunofluorescence of the AVP peptide alone shows AVP-peptidergic fibers in anterior-to-posterior BNST. ac, anterior commissure; BNST_AM,_ anteromedial BNST; BNST_OV,_ oval nucleus of the BNST; CP, caudate putamen; IC, internal capsulae; LV, lateral ventricle; opt, optic track; 3^rd^V, third ventricle.

AVP-immunoreactive fibers were present throughout the anterior-to-posterior BNST, with the greatest expression in the anteromedial BNST along the anterior-posterior axis, and in the more posterior divisions (starting at bregma −0.30). Relatively dense expression was also observed in the sub-commissural BNST. In the BNST_DL_, most AVP fibers were located next the internal capsule and below the lateral ventricle, whereas moderate density was found in the BNSTov (Figures 5H–5H”’).

**Figure 6.**
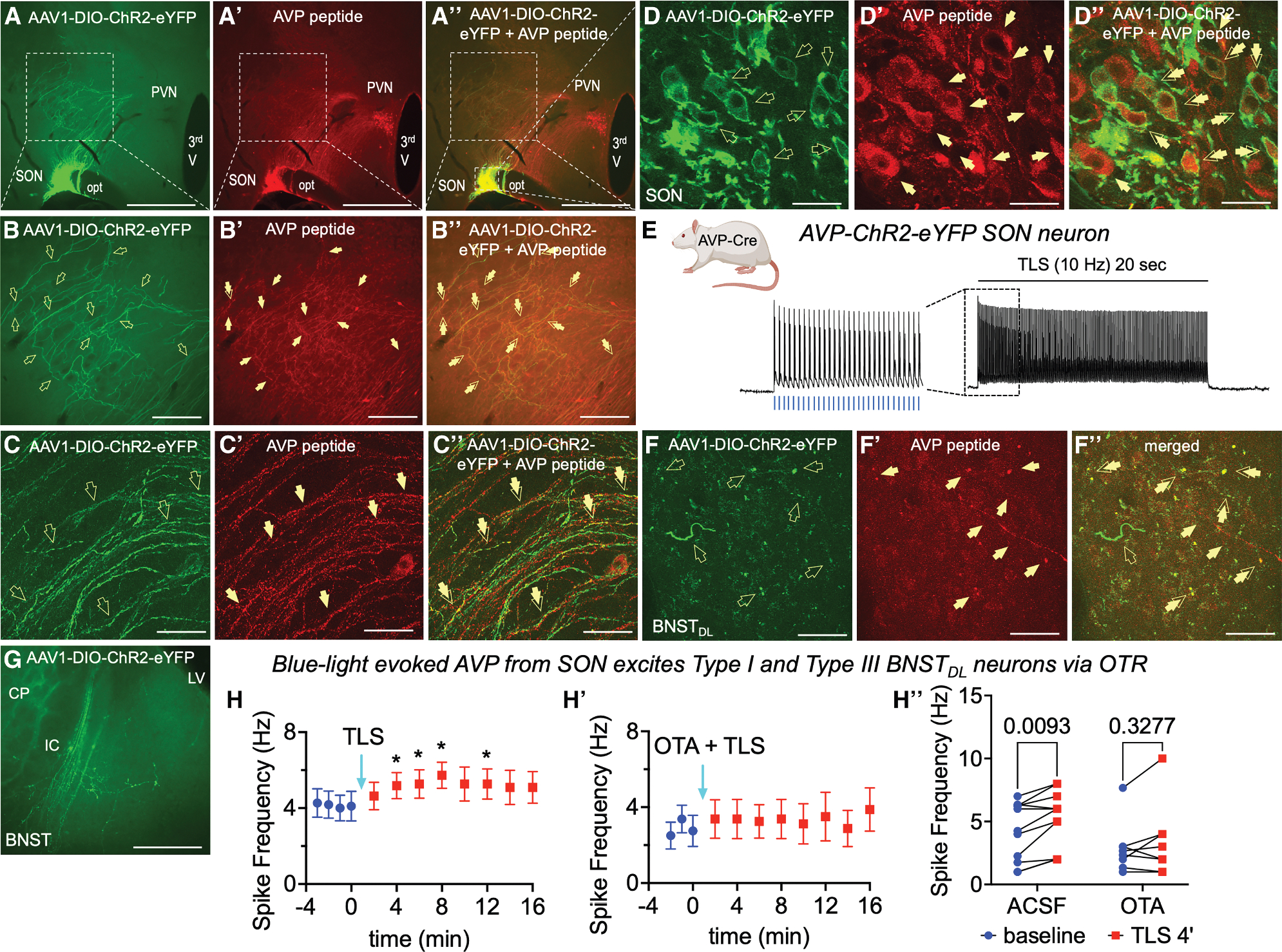
TLS of AVP-SON fibers excites BNST_DL_ neurons in an OTR-dependent fashion (A) AVP-Cre rats were injected with AAV-EF1a-DIO-ChR2-eYFP (green) in the SON, showing co-expression of eYFP-ChR2 and AVP-peptide (Alexa-Flour 594, red), which was not observed in the PVN (A-A″, original magnification ×4; scale bar, 100 μm). (B–D″) High co-expression of ChR2-eYFP (open arrows) and AVP-peptide (closed arrows) in SON-originating fibers, which ascend dorsolaterally toward IC (original magnification ×20; scale bar, 50 μm) (original magnification ×60; scale bar, 10 μm). Confocal images (original magnification ×60) show co-localization of ChR2-eYFP (open arrows) and AVP (closed arrows) in the soma and dendrites of SON neurons (scale bar, 10 μm). (E–F″) In the BNST_DL_, confocal images show ChR2-eYFP-expressing (open arrows) and AVP-containing fibers (closed arrows) converging on boutons which co-express ChR2-eYFP and AVP peptide (double arrows; scale bar, 10 μm). Action potentials recorded from ChR2-eYFP-expressing SON neurons show burst firing following TLS (10 Hz), where each pulse of blue light evokes action potential. (G) High expression of ChR2-eYFP-expressing processes and fibers originating from the SON, with fluorescent boutons (presumptive release sites) is seen in the BNST from brain slice processed after electrophysiology (scale bar, 100 μm). (H) In the BNST_DL_, action potentials frequency was recorded, before and after TLS of hypothalamic ChR2-eYFP fibers in response to an 80-pA current, with and without OTA. In types I and III BNST_DL_ neurons, there was a significant TLS effect on firing frequency (*p* = 0.0459), but not with OTA (*p* = 0.2863) (H′). Comparison of firing frequency with or without OTA, showed a significant TLS effect, but not with OTA. CP, caudate putamen; IC, internal capsulae; LV, lateral ventricle; 3^rd^V, third ventricle. *p < 0.05.

**Figure 7.**
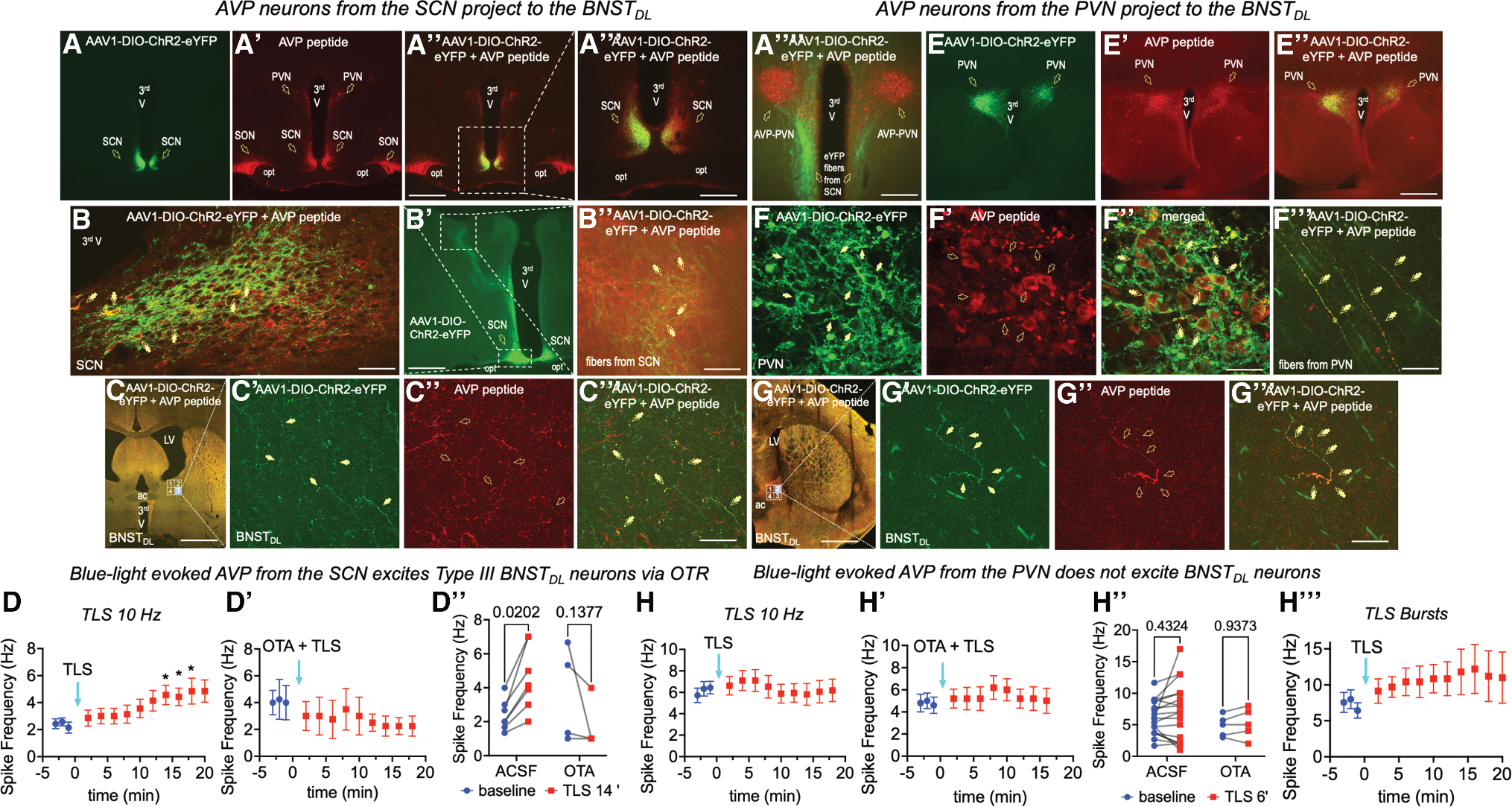
TLS of AVP-SCN fibers excites BNST_DL_ neurons in an OTR-dependent fashion AVP-Cre rats were injected with AAV-EF1a-DIO-ChR2-eYFP (green) in the SCN (A–B″) or PVN (E-G‴) and AVP-peptide was visualized with Alexa-Flour 594 (red). Rats injected in the SCN co-express eYFP-ChR2 and AVP-peptide in the SCN, but not SON or PVN at an original magnification of ×4 (A–A″; scale bar, 100 μm) and an original magnification of ×10 (A‴; scale bar, 50 μm). High co-expression of ChR2-eYFP and AVP peptide in the SCN (B) (double-arrows; an original magnification of ×60; scale bar, 10 μm) and SCN-originating fibers, which ascend dorsally toward the PVN (A‴’ and B′; original magnification ×10), and travel at a straight angle toward posterior BNST (B′) (original magnification ×10), where they co-express AVP-peptide (B″) (double-arrows; original magnification ×20; scale bar, 20 μm). In the BNST_DL_, ChR2-eYFP-(open arrows) and AVP-fibers/processes (closed arrows) co-express (double arrows; original magnification ×60; scale bar, 10 μm) (C–C‴). Action potentials frequency was recorded from BNST_DL_ neurons, before and after TLS (10 Hz) of SCN-ChR2-eYFP fibers, in response to an 80-pA current, with/without OTA. In type III neurons, there was a significant TLS effect (*p* = 0.0251) (D), but not with OTA (*p* = 0.4261) (D′). When firing following TLS was analyzed with/without OTA, a significant TLS-OTA interaction emerged (*p* = 0.0059), with increased firing following TLS but not with OTA (D″). Rats injected in the PVN co-express eYFP-ChR2 and AVP in the PVN, but not SON (E–E″) (original magnification ×4; scale bar, 100 μm). High co-expression of ChR2-eYFP (closed arrows) (F) and AVP (open arrows) (F′) in the PVN neurons (double arrows) (F″) (confocal image; original magnification ×60; scale bar, 10 μm) and PVN-originating fibers (F‴’) (original magnification ×60). In the BNST_DL_, ChR2-eYFP-fibers and processes (G′) co-express AVP (G″) (double arrows; original magnification ×60; scale bar, 10 μm) (G‴). Spiking frequency (Hz) of BNST_DL_ neurons, before and after TLS (10 Hz) of PVN-ChR2-eYFP fibers in response to an 80-pA current, with and without OTA. In types I and III neurons, there was no TLS effect (*p* = 0.5533) (H), and no TLS effect with OTA (*p* = 0.2920) (H′). No treatment or TLS effect emerged when firing was analyzed with/without OTA (H″). Modified protocol with repeated TLS bursts did not increase BNST neurons’ firing (H‴). ac, anterior commissure; CP, caudate putamen; LV, lateral ventricle; opt, optic track; 3^rd^V, third ventricle. *p < 0.05.

AVP-Cre rats were injected bilaterally into the SON, SCN, or PVN with a Cre-dependent pAAV-hSyn-FLEx-mGFP-2A-synaptophysin-mRuby and perfused (*n* = 8), or with pAAV-EF1a-double-floxed-hChR2(H134R)-eYFP-WPRE-HGHpA and used for optogenetics during electrophysiology. All brains were examined *post hoc* for neuronal projections from AVP neurons to the BNST_DL_ (SON *n* = 28; SCN *n* = 30; PVN *n* = 23). Fidelity of the Cre-expression was confirmed in the cell bodies and processes of AVP neurons in the SON, SCN, and PVN, where synaptophysin-mRuby (Figures 5A–5B”’ and 5D–5E”’) or EF1a-ChR2-eYFP-expressing neurons and processes co-expressed AVP-peptide (Figures 6A–D” and 7A–F”’). Fibers and processes expressing mRuby or eYFP were seen to ascend from the SCN neurons and traversed dorsally along the third ventricle, innervating the anterior hypothalamus and the PVN, including the PVN_AVP_ neurons (Figure 7A””), and then continued via a right angle toward the posterior BNST and dorsally toward the lateral septum (Figure 7B”). From the SON, mRuby or eYFP-expressing fibers and processes ascended via a lateral-dorsal route toward the hypothalamic accessory nuclei and the PVN (Figures 6A–6C”), and then laterally along the internal capsule toward the posterior BNST (Figure 6G). From the PVN, eYFP-expressing fibers and processes descended toward SON, and traveled via a lateral route toward the internal capsule (Figure 7E). The eYFP-expressing fibers and processes originating from the SON (Figures 6B–6C”), SCN (Figure 7B’ and 7B”), and PVN (Figure 7F”) co-expressed AVP peptide. Notably, both synaptophysin-mRuby and EF1a-ChR2-eYFP-expressing fibers originating from the SON (Figures 5F–5F”’ and 6F–F”), SCN (Figures 5C–5C”’ and 7C–7C”’), and PVN (Figures 7G–7G”’), were found in the BNST_DL_, and a subset of these fibers co-expressed AVP peptide. We also conducted an additional control experiment to exclude a possibility of signal bleeding and overlap. Confocal images of mRuby signal alone confirmed the presence of synaptophysin-mRuby fibers in the BNST_DL_, originating from the SCN (Figures 5G–5G”) or SON (Figures 5G” and 5G”’).

### Optogenetically evoked AVP release from hypothalamic fibers excites BNST_DL_ neurons

We first established an optogenetic protocol for hypothalamic peptide release based on the well-characterized cellular effects of OT in the BNST^38^ (Figure S2). To determine functional peptidergic projections from hypothalamic AVP neurons to the BNST_DL_, action potentials frequency (Hz) was recorded from type I and III BNST_DL_ neurons before and after tetanic light stimulation (TLS; 10 Hz) of hypothalamic ChR2-eYFP fibers in BNST slices. The recordings, conducted with synaptic transmission blockers, tested whether optogenetically evoked AVP mimics the excitatory effects of exogenous AVP in response to 80-, 100-, and 120-pA current injections. In separate rats, a modified protocol mimicked the AVP-PVN neuron firing, delivering four bursts of blue light pulses every 30 s (in response to 40-, 60-, and 80-pA currents). ChR2-eYFP fibers from the SON, SCN, and PVN were stimulated separately, with and without OTA. At least three or four pre-TLS responses per current were collected per neuron and averaged as a baseline for RM analyses. Only rats with strong bilateral, robust unilateral, or moderate bilateral ChR2-eYFP expression in the hypothalamus were included in the analysis.

#### AVP from the SON excites type I and type III BNST_DL_ neurons via OTR

We first confirmed that TLS (10-ms pulses, 10 Hz) reliably evoked action potentials in eYFP-ChR2-expressing AVP-SON neurons (Figures 6D and 6E). After TLS of ChR2-expressing fibers in the BNST_DL_, type I neurons showed a current effect on spike frequency, *p* = 0.0283, F(1.234, 7.404) = 6.912, *n* = 7 neurons, 5 rats, 2-way RM ANOVA unless stated otherwise, but no TLS effect, *p* = 0.5131, F(1.630, 9.777) = 0.6497, or interaction, *p* = 0.3164, F(1.918, 11.51) = 1.267. Similarly, type III neurons showed current effect, *p* = 0.0123, F(0.5618, 2.247) = 58.26, *n* = 4, 4 rats, but no TLS effect, *p* = 0.2023, F(1.405, 5.621) = 2.137, or interaction, *p* = 0.5631, F(1.782, 6.147) = 0.5937. However, when type I and type III neurons were combined, TLS increased spiking, *p* = 0.0459, F(3.166, 31.66) = 2.936, ANOVA, with *post hoc* effects at 4 min (*p* = 0.0199), 6 min (*p* = 0.0255), 8 min (*p* = 0.0245), and 12 min (*p* = 0.0255) (Figure 6H), in response to 80 pA.

When the OTR antagonist OTA was applied before TLS, there was no effect on firing frequency in response to 80-pA current, *p* = 0.2863, F (3.286, 23.00) = 1.339, *n* = 3 type I, 3 rats and *n* = 5 type III, 4 rats (Figure 6H’). A two-way ANOVA comparing the TLS and OTA plus TLS groups confirmed a significant TLS effect, *p* = 0.0402, F(3.825, 65.03) = 2.701, *n* = 19 neurons, with increased firing at 4 min (*p* = 0.0285), 6 min (*p* = 0.0369), 8 min (*p* = 0.0354), and 12 min (*p* = 0.0369). However, TLS had no effect at any time point in the OTA plus TLS group. As the strongest TLS effect occurred at 4 min, we compared this firing frequency vs. baseline, with and without OTA. There was a TLS effect, *p* = 0.0055, F(1, 17) = 10.09, *n* = 19, but no treatment effect, *p* = 0.1705, F(1, 17) = 2.048, or interaction, *p* = 0.3088, F(1, 17) = 1.101. *Post hoc* analysis showed that TLS increased firing of BNST_DL_ neurons (*p* = 0.0093) but had no effect when OTRs were blocked (*p* = 0.3277) (Figure 6H”).

#### AVP from the SCN excites type III BNST_DL_ neurons via OTR

In type I BNST_DL_ neurons from rats injected with ChR2-eYFP in the SCN, there was a current effect on spike frequency, *p* = 0.0001, F(1.338, 10.70) = 28.60, *n* = 9, 9 rats, but no TLS effect, *p* = 0.3718, F(1.888, 15.10) = 1.045, or interaction, *p* = 0.2841, F(3.111, 24.89) = 1.340. Similarly, firing in response to 80 pA showed no TLS effect, *p* = 0.5633, F(2.918, 23.34) = 0.6903, *n* = 9. However, type III BNST_DL_ neurons showed a trend toward TLS effect on spike frequency, *p* = 0.0586, F(1.383, 8.297) = 4.443, *n* = 7, 6 rats, and a current effect, *p* = 0.0088, F(1.051, 6.306) = 13.70, with no interaction, *p* = 0.1178, F(2.840, 17.04) = 2.286. *Post hoc* analysis showed a significant TLS effect at 12 min (vs. baseline *p* = 0.0364), 14 min (*p* = 0.0154), 16 min (*p* = 0.0289), and 20 min (*p* = 0.0243) in response to 80 pA injection. A separate analysis of 80-pA responses confirmed a significant TLS effect, *p* = 0.0251, F(1.739, 10.43) = 5.611, *n* = 7, ANOVA, with the same time-dependent increases (Figure 7D).

With OTA, TLS had no effect on type III neuron firing at 80 pA, *p* = 0.4261, F(1.945, 5.836) = 0.9831, ANOVA, *n* = 4, 4 rats (Figure 7D’). A two-way ANOVA comparing TLS effects with and without OTA (at 80 pA) showed a TLS-OTA interaction, *p* = 0.0059, F(1, 9) = 12.84, *n* = 11, but no TLS, *p* = 0.7535, F(1, 9) = 0.1048, or treatment effect, *p* = 0.4243, F(1, 9) = 0.7004. *Post hoc* analysis showed that TLS increased type III neuron firing at 14 min (*p* = 0.0202), but this effect was absent with OTA (*p* = 0.1377) (Figure 7D”).

#### AVP from the PVN does not excite BNST_DL_ neurons

In types I and III BNST_DL_ neurons from PVN-ChR2-eYFP rats, there was a current effect on spike frequency, *p =* 0.0310, F(1.597, 27.15) = 4.307, but no TLS effect, *p* = 0.5533, F(1.596, 27.13) = 0.5329, or interaction, *p* = 0.4074, F(2.403, 35.30) = 0.9579, *n* = 18 neurons, 10 rats. Analysis of spiking in response to 80 pA showed no TLS effect, *p* = 0.3825, F(1.648, 25.64) = 0.9540, *n* = 18 (Figure 7H’), and no effect in a presence of OTA, *p* = 0.2920, F (1.668, 6.671) = 1.457, *n* = 5 neurons, 4 rats (Figure 7H”). When comparing firing after TLS with and without OTA, no treatment effect, *p* = 0.3456, F (1,21) = 0.9311, TLS effect, F(1, 21) = 0.7087, *p* = 0.4094, or interaction, *p* = 0.7901, F(1, 21) = 0.07267, *n* = 23, emerged (Figure 7H”’).

When a modified protocol was applied to mimic a AVP-PVN neurons firing pattern,^45–48^ repeated TLS bursts revealed a current effect on firing-frequency of type I and III BNST_DL_ neurons, *p* = 0.0136, F(1.013, 6.079) = 11.73, *n* = 7 neurons, 3 rats, but no TLS effect, *p* = 0.3217, F(1.983, 11.90) = 1.248, or interaction, *p* = 0.4875, F (2.024, 9.716) = 0.7777. There was no TLS effect, *p* = 0.3025, F (1.874, 9.745) = 1.346, in response to 40 pA current. A paired *t* test at 10 min post-TLS showed a trend (*p* = 0.0872, *n* = 7) (Figure 7H”’), but despite four of seven neurons exhibiting high to moderate firing increases, the TLS effect was not significant (*p* = 0.1996, *n* = 4).

## DISCUSSION

We provide evidence that AVP-OT crosstalk via OTR-expressing BNST_DL_ neurons modulates anxiety-like behavior. Exogenous AVP directly and robustly excited types I and III neurons in the BNST_DL_, a key region for fear processing and vigilant threat monitoring,^24–27^ and this excitatory effect required OTR transmission. Specifically, OTR, but not V1aR or V1bR antagonists, blocked the excitatory effects of AVP on intrinsic membrane properties and firing frequency of BNST_DL_ neurons. In a subset of type III BNST_DL_ neurons, V1aR activation by the selective agonist FE201874, which also blocks OTR,^49^ moderately increased firing without affecting membrane properties. Excitatory effects of OTR have been shown in the cortex, hippocampus, and the BNST,^38,50–52^ while both OTR and V1aR show excitatory effects in the CeA, although on different neuronal populations.^50,53,54^ AVP and OT differ by only two amino acids, and the cross-talk between these neuropeptides and their receptors is well documented.^55–57^ OTR and V1aR, both centrally located G protein-coupled receptors,^58^ share 85% structural homology,^59,60^ while V1bR shares 40% identity with V1aR.^10^ Although OT and AVP have a similar affinity for OTR, OT has a greater affinity for OTR than for V1aR or V1bR.^61,62^

The stronger excitatory response of type III neurons to AVP, which acts via OTR and V1aR, compared with OT or the selective OTR agonist, TGOT,^63,64^ may be explained by the differences in their affinities toward the receptors and the combined action of these receptors. The I/O curves differ for AVP vs. OT, with OT inducing robust excitation at lower currents, while AVP shifts the I/O curve in parallel across all currents. This suggests that, while V1aR activation alone has a modest effect, with OTR activation, it sustains the excitatory effect of AVP in type III neurons. In contrast, while AVP activates both OTR and V1aR in type III BNST_DL_ neurons to robustly increase excitability, in type I neurons, V1aR provides tonic inhibition to a subset of neurons, despite the excitatory effect of OTR. The inhibition was unmasked by the excitatory effect of V1aR antagonist, consistent with prior reports of central V1aR-mediated inhibition.^65^ However, OTR remains the key player in regulating BNST_DL_ neuron excitability and BNST_DL_-dependent behaviors.

In many brain regions, OT-AVP crosstalk has limited physiological and behavioral impacts because OTR and V1R are often located in separate circuits. In such cases, their effects depend on site-specific receptor expression.^66,67^ However, BNST expresses both OTR^17,38^ and V1aR^68^ and we show cell-type-specific functional expression of these receptors in types I and III neurons, aligning with our recent study showing post-synaptic expression of OTR in these cell types.^38^ We confirmed AVP’s excitatory effect on fluorescent OTR-BNST_DL_ neurons, with most identified as type III. Previous single-cell PCR studies have shown that type III BNST_DL_ neurons express OTR, CRF, and STEP mRNA.^29^ Using CRF-Cre transgenic rats, we showed that CRF-mCherry-BNST_DL_ neurons, which exhibit electrophysiological properties of type III, are directly excited by both AVP and TGOT. While the co-expression of CRF and OTR in BNST_DL_ neurons was first described in 2011,^29^ this study provides the first functional evidence of OTR activation directly exciting CRF-BNST_DL_ neurons. Consistent with *in situ* hybridization and OTR-binding studies^17,18,69^ identifying rodent BNST as a high OTR-expressing region, we observed numerous OTR neurons throughout the BNST of OTR-Cre rats. Recent single-cell RNA sequencing analysis in mice revealed OTR expression in diverse GABAergic neurons in the BNST.^31^ Here, we found that OTR-BNST_DL_ neurons co-express either PKCδ or STEP, two enzymes expressed by distinct BNST_DL_ neurons.^70^ STEP is uniquely expressed by CRF neurons in the oval nucleus of the BNST_DL_ and exclusively marks type III BNST_DL_ neurons on a single-cell level.^33^

While our study focused on the BNST_DL_, OTR neurons are also present in the posterior BNST, and the high density of AVP fibers suggests that this region may also underlie the relevant effects of AVP on BNST activity and behaviors. The identified markers are not exclusive, as OTR can also be expressed in astrocytes, as seen in the CeA.^71^ However, unlike the BNST_DL_, OTR and V1aR in CeA are found on separate neuronal populations, with opposing effects on medial CeA output neurons and fear behaviors.^16,32,50^ Thus, type III BNST_DL_ neurons form a unique population in the extended amygdala, where OTR and V1aR signaling converge to increase excitability in response to AVP. However, strong AVP innervation of anteromedial BNST, where there are fewer OTR neurons, suggests that AVP may also exert direct effects independent of OTR.

OTR transmission in the extended amygdala plays an intricate role in fear and vigilance. While OTR activation in the CeA reduces contextual fear,^32,72,73^ in the basolateral amygdala, it enhances fear discrimination toward discrete cues predicting danger.^74^ Our previous studies showed that blocking OTR in the BNST_DL_ impaired both the acquisition and recall of cued fear in FPS.^26^ Notably, it also reduced the ratio of cued to non-cued fear, suggesting that OTR-BNST_DL_ transmission strengthens fear responses to discrete threats while reducing threat-induced anxious arousal.^26,37^ Similarly, systemic or intracerebroventricular OT reduces non-cued fear in FPS.^75–77^ In this study, chemogenetic silencing of OTR-BNST_DL_ neurons delayed the extinction of non-cued fear in FPS. Non-cued fear reflects heightened startle that occurs between cue presentations, emerging only after the cue is being presented.^26,76^ As such, it represents a cue-induced vigilant state or anxious arousal, which is independent from contextual fear.^43^ These findings underscore the necessity of OTR and OTR-BNST_DL_ neurons in enhancing fear learning toward signaled threats while reducing threat-induced anxious arousal.

In fear-conditioned rats, silencing OTR-BNST_DL_ neurons reduced open-arm time in the EPM, indicating their necessity for the anxiolytic-like effect. This aligns with extensive literature showing that OTR activation, especially in stressed animals, has anxiolytic effects.^7,8,78^ BNST_DL_ activity mediates hypervigilance and anticipatory anxiety in response to unpredictable or un-signaled threats.^25,79,80^ While the oval nucleus of the BNST was identified as an anxiogenic node, this was based on optogenetic manipulations in dopamine-D1 receptor Cre mice,^81^ making the effect specific to D1R-neurons in the BNST_OV_, which is otherwise a heterogeneous nucleus with diverse neurons. Although OTR-BNST_DL_ neurons play an anxiolytic role, seemingly contrasting with the overall anxiogenic function of the BNST, we previously demonstrated that OTR activation excites BNST_DL_ interneurons, increasing inhibitory synaptic transmission and inhibiting type II BNST_DL_ output neurons. This leads to an overall inhibitory effect on BNST_DL_ projections to the CeA,^38^ which was previously shown to reduce open arm exploration in the EPM.^82^

AVP and OT influence fear and anxiety-like behaviors, and are often labeled as anxiogenic and anxiolytic peptides, respectively. The anxiogenic label for AVP originates from studies on AVP-deficient Brattleboro rats, which showed reduced vigilance in open spaces and reduced contextual fear.^83–85^ Consistently, systemic V1aR activation reduced exploration of open arms in the EPM, while V1aR antagonism or knockout increased it.^19,86–88^ However, V1aR activation in the lateral septum and posterior BNST can increase EPM exploration and reduce defensive behaviors,^30,89^ suggesting a more nuanced role of V1aR in fear modulation. Notably, the commonly used V1aR-selective antagonist, the Manning compound (d(CH_2_)_5_[Tyr(Me)^2^]AVP), although not binding V1bR or V2R, acts as a potent OTR antagonist.^90^ Given the abundant expression of both OTR and V1R in the hypothalamus and extended amygdala, AVP’s effects via OTRs may be underestimated.^19,20^ Our findings show that AVP infusion into the BNST_DL_ does not alter behavior in the FPS or EPM (Figure S1). Although unexpected, our previous study also found that, while OTR blockade affects fear in the FPS, infusing exogenous OT has little or inconsistent effect.^26^ Since OT is released in the BNST_DL_ during fear conditioning,^37^ this might explain why additional OT does not further impact fear behavior. Future studies will determine whether AVP from distinct hypothalamic sources is released in the BNST during fear conditioning. Our finding that AVP increased BNST_DL_ neuronal excitability, requiring OTR signaling (with V1aR showing moderate effects), suggests that, rather than working in opposition, OTR and V1aR, modulated by specific hypothalamic inputs, work in tandem to regulate BNST activity and behaviors.

Previous studies have shown that the rat BNST_DL_ receives OT inputs from the PVN,^29,32,37^ which we confirmed here using an optogenetic approach. We also showed that the BNST_DL_ receives AVP inputs from the SON, SCN, and PVN. Optogenetic stimulation of AVP terminals from the SCN or SON increased types I and III BNST_DL_ neurons’ excitability via OTR. The lack of TLS effect on PVN-BNST input was unexpected, but given the AVP-PVN role in stress axis regulation, AVP release in non-stressed rats might not be impacted. Cell-selective genetic manipulations have provided insights into the role of specific hypothalamic and extra-hypothalamic AVP neurons to defensive behaviors. For example, eliminating AVP-PVN or AVP-SCN neurons in AVP-Cre mice reduced open-arm exploration in the EPM,^91^ whereas removing AVP cells in the posterior BNST had no effect. These findings suggest that hypothalamic AVP neurons diminish vigilance and promote open-arm exploration in the EPM. It is noteworthy that both the AVP-SCN^91^ and the OTR-BNST_DL_ neuron activity increases rodents’ open-arm time in the EPM. Thus, during periods of greater AVP-SCN neuron activity, such as early light phase,^1,92–94^ the SCN-BNST projection may be primed for AVP release in response to light-cues, activating OTR-BNST_DL_ neurons to reduce anxiety. Our study reveals cell-type- and receptor-specific modulation of BNST_DL_ neuron activity via distinct hypothalamic AVP projections. While both OT and AVP excite type III OTR-BNST_DL_ neurons, their effects differ in receptor involvement (OTR vs. OTR and V1aR), leading to differences in the excitatory responses’ magnitude. These findings suggest that changes in physiological states favoring OT vs. AVP release (e.g., lactation vs. thirst/salt intake) will scale excitatory responses of type III OTR-BNST_DL_ neurons, fine tuning their I/O dynamics, and providing them with greater flexibility to specific neurohypophysial inputs.

### Limitations of the study

First, female rats were not included. While future studies will examine AVP-OT crosstalk in female BNST excitability and behavior, a detailed analysis of both sexes was beyond this study’s scope. Combining males and females in the same groups could obscure the significant role the OTR and V1aR^28,95^ play in sex differences and similarities in stress-related behaviors, including non-cued fear.^43^ Second, while we confirmed hypothalamic projections, we cannot rule out the possibility that local AVP neurons from the posterior BNST, known for their sex-dependent function and expression (high in male rats),^96^ may also project to the BNST_DL_.^22^ Finally, although this study focused on postsynaptic OTR and V1R function, the use of synaptic transmission blockers prevented us from examining whether V1aR, like OTR,^32,38^ has a distinct effect on synaptic activity in the BNST_DL_.

### RESOURCE AVAILABILITY

#### Lead contact

Requests for further information and resources should be directed to and will be fulfilled by the lead contact, Joanna Dabrowska, PhD, PharmD, Center for the Neurobiology of Stress Resilience and Psychiatric Disorders, Discipline of Cellular and Molecular Pharmacology, The Chicago Medical School, Rosalind Franklin University of Medicine and Science, 3333 Green Bay Road, North Chicago, IL, 60064 Tel: 847–578-8664 (joanna.dabrowska@rosalindfranklin.edu).

#### Materials availability

This study did not generate any new or unique reagents or materials.

#### Data and code availability

- Electrophysiology data (MATLAB files) and behavioral data (CSV, Comma Separated Values files) is available at ndi-cloud.com/datasets/67f723d574f5f79c6062389d (https://doi.org/10.63884/ndic.2025.jyxfer8m), neuroscience and physiology repository with a searchable database.^97^
- The custom MATLAB code used for electrophysiology data acquisition and analysis created by Dr. Niraj Desai (NIH) are available on https://doi.org/10.5281/zenodo.15238413.
- Original microscopy data reported in this paper will be shared by the lead contact upon request.

## STAR★METHODS

### EXPERIMENTAL MODEL AND STUDY PARTICIPANT DETAILS

Male wild-type Sprague-Dawley rats (SD, Envigo, RRID:MGI:5651135, Chicago, IL; 240–300 g, 60–90 days old), male transgenic OTR-Cre (Cre-recombinase (Cre) under OTR promoter), CRF-Cre (Cre under CRF promoter), and AVP-Cre (Cre under AVP promoter) rats (220–400 g, all 60–90 days old) were housed in groups of three on a 12-h light/dark cycle (light 7 a.m. to 7 p.m.) with free access to water and food. Upon arrival from Envigo, wild-type SD rats were habituated to the environment for one week before the experiments began. OTR-Cre^39,40^ and AVP-Cre transgenic (knock-in) rats were originally generated and kindly provided by Dr. Valery Grinevich (Heidelberg University, Germany),^98^ and CRF-Cre rats^99,100^ were created and kindly provided by Dr. Robert Messing (University of Texas, Austin) and bred at the biological resources facility (BRF) at Rosalind Franklin University of Medicine and Science (RFUMS). Experiments were performed in accordance with the guidelines of the NIH and approved by the Animal Care and Use Committee at RFUMS.

No human subjects were used in the study.

### METHOD DETAILS

#### Drug preparation and pharmacological manipulations

The following compounds were used for electrophysiology: arginine (Arg^8^)-vasopressin (AVP; H-Cys-Tyr-Phe-Gln-Asn-Cys-Pro-Arg-Gly-NH_2_ (disulfide bond); 0.2 μM for electrophysiology, 10 ng/0.5 μL per hemisphere for behavioral testing, Bachem Americas, Torrance, CA; catalog #4012215); oxytocin (OT; H-Cys-Tyr-Ile-Gln-Asn-Cys-Pro-Leu-Gly-NH2 (disulfide bond, acetate salt), 0.2 μM, Bachem Americas, Torrance, CA, #4016373); a selective rat V1aR agonist FE201874 (0.2–0.4 μM, generously provided by Ferring Pharmaceuticals, San Diego, CA),^49^ a selective and potent OTR agonist TGOT ([Thr,^4^Gly^7^]-oxytocin (0.4 μM, Bachem Americas, Torrance, #4013837), and V1bR agonist d[Cha4]-AVP (1 μM, GlpBio Technology Inc, Montclair, CA, #GC1659).^3,101^ The following antagonists were used: a V1aR/OTR antagonist (d(CH_2_)_5_^1^,Tyr(Me),^2^Arg^8^)-vasopressin, Manning compound^90^; 1 μM, Tocris, Bio-Techne Corporation; MN, #**3377**); selective OTR antagonist (OTA; d(CH2)5(1), D-Tyr(2), Thr(4), Orn(8), des-Gly-NH2(9)]-Vasotocin trifluoroacetate salt^90^; 0.4 μM, Chemical Repository, #V-905 NIMH); selective V1aR antagonist SR49059 (2*S*)-1-[[(2*R*,3*S*)-5-Chloro-3-(2-chlorophenyl)-1-[(3,4-dimethoxyphenyl)sulfonyl]-2,3-dihydro-3-hydroxy-1*H*-indol-2-yl]carbonyl]-2-yrrolidinecarboxamide; 5 μM, Tocris, Bio-Techne Corporation, #2310); and a V1bR antagonist Nelivaptan ((2*S*,4*R*)-1-[(3*R*)-5-Chloro-1-[(2,4-dimethoxyphenyl)sulfonyl]-2,3-dihydro-3-(2-methoxyphenyl)-2-oxo-1*H*-indol-3-yl]-4-hydroxy-*N*,*N*-dimethyl-2-pyrrolidinecarboxamide; 1 μM, Tocris, Bio-Techne Corporation, #6195). To block glutamatergic transmission, we used AMPA and kainate receptor antagonist *6-Cyano-7-nitro-quinoxaline-2,3-dione disodium salt (CNQX,* 10 μM, Tocris, Bio-Techne Corporation, #1045). To block *NMDA receptors,* we used D-2-Amino-5-phosphopentanoic acid (D-AP5, 50 μM, Tocris, Bio-Techne Corporation, #0106). To block GABA-A receptors we used picrotoxin (PTX, 25 μM, Tocris, Bio-Techne Corporation, #1128). The designer receptor exclusively activated by designer drugs (DREADD) ligand clozapine-N-oxide (CNO), 8-Chloro-11-(4-methyl-4-oxido-1-piperazinyl)-5*H*-dibenzo[*b*,*e*][1,4]diazepine (CNO, Tocris, Bio-Techne Corporation, #4936) was used at 20 μM for chemogenetics manipulations during electrophysiological recordings and at 2 mg/kg for behavioral experiments. All drug stock solutions were dissolved in sterile deionized water, unless stated otherwise, and stored at −20°C until use. The day of the experiment, drugs for electrophysiology were diluted into artificial cerebrospinal fluid (aCSF) and applied to bathe solution. CNO was diluted into sterile saline and injected intraperitoneally (i.p.) at 2 mg/kg

#### *In vitro* whole-cell patch-clamp electrophysiology

Slice preparation and electrophysiological recordings were performed as before.^38^ Rats were deeply anesthetized by inhalation of isoflurane USP (Patterson Veterinary, Greeley, CO, USA). After decapitation, the brain was rapidly removed from the cranial cavity, and 300-μm-thick coronal slices containing the BNST were prepared in ice-cold cutting solution (saturated with 95% O_2_/5% CO_2_) containing in mM: 122.5 NaCl, 3.5 KCl, 25 NaHCO_3_, 1 NaH_2_PO_4_, 0.5 CaCl_2_^.^2H_2_O, 20 D-glucose, 3 MgCl_2_ 6H_2_O, and 1 ascorbic acid (pH 7.4, 290–300 mOsm). The slices were prepared using a Leica vibratome (VT1200; Leica, Wetzlar, Germany), incubated for 30 min at 34°C, and subsequently transferred to room temperature for 1 h before the recordings began. Next, the slices were transferred to a recording chamber perfused with oxygenated aCSF at a rate of 2–4 mL/min containing in mM: 122.5 NaCl, 3.5 KCl, 25 NaHCO_3_, 1 NaH_2_PO_4_, 2.5 CaCl_2_^.^2H_2_O, 20 D-glucose, and 1 MgCl_2_^.^6H_2_O (pH 7.4, 290–300 mOsm), saturated with 95% O_2_ and 5% CO_2._ The aCSF was warmed to 30°C–34°C by passing it through a feedback-controlled in-line heater (TC-324C; Warner Instruments, Hamden, CT). The cell bodies of BNST_DL_ neurons were visualized using infrared differential interference contrast (IR-DIC) optics with an upright microscope (Scientifica Slice Scope Pro 1000, Clarksburg, NJ). Neurobiotin (0.1%, Vector Laboratories, Burlingame, CA) was added to the internal solution of the recording pipette to confirm cell location. After recording, slices were immersed in 10% formalin (Fisher Scientific, SF98–4), washed three times (10 min each) in phosphate-buffered saline (PBS 0.05 M), and incubated with streptavidin-Alexa Fluor 594 or Alexa Fluor 488 conjugate (dilution 1:2000, Invitrogen ThermoFisher Scientific, S32356 and S11223, respectively) at room temperature for 2 h, followed by three washes in PBS and one wash in phosphate buffer (PB 0.05 M) (10 min each). All slices were mounted using Mowiol with antifade reagent (Sigma-Aldrich, 81381), and coverslips were applied before visualization with fluorescent microscopy (Nikon eclipse N*i*, Nikon Instruments Inc.)

Electrophysiology recordings were made using Axon pCLAMP11 (Molecular Devices, RRID:SCR_011323), Multiclamp 700B amplifiers (Axon Instruments, Union City, CA); current-clamp signals were acquired at 10kHz with a 16-bit input-output board NI USB-6251 (National Instruments, Austin, TX), using custom MATLAB scripts (Math-Work, Natick, MARRID:SCR_001622) written by Dr. Niraj Desai (NIH; Bethesda; USA).^102^ The access resistance (Ra) was monitored, and recordings were terminated if Ra changed >15%. Electrophysiological measurements were carried out 10–15 min after reaching the whole-cell configuration. The input resistance was calculated from steady-state voltage responses upon negative current injections (pulses of 450–1000 ms). Whole-cell patch-clamp recordings were made from BNST_DL_ neurons using glass pipettes (4–8 MΩ) pulled from thick-walled borosilicate glass capillaries with a micropipette puller (Model P-97; Sutter Instrument, Novato, CA) filled with a solution containing the following in mM: 135 potassium gluconate, 2 KCl, 3 MgCl2.6H_2_O, 10 HEPES, 5 Na-phosphocreatine, 2 ATP-K, and 0.2 GTP-Na (pH 7.3 adjusted with KOH, osmolarity 300–305 mOsm; potentials were not corrected for a liquid junction potential), as before.^38^

To study the effects of AVP on intrinsic membrane properties and intrinsic excitability, the recordings were performed in the presence of synaptic transmission blockers: the AMPA receptor antagonist CNQX, the NMDA receptor antagonist D-AP5, and GABA-A receptor antagonist, PTX. To verify the receptor involved in the effects of AVP, in separate experiments, AVP was perfused in the presence of the V1aR/OTR antagonist, selective OTR antagonist OTA, selective V1aR antagonist SR49059, or selective V1bR antagonist Nelivaptan. Each antagonist was first applied alone for 10 min and then maintained during AVP application for another 12–15 min, followed by a 12–15-min drug-free washout in all whole-cell patch-clamp experiments.

#### Electrophysiological characterization of BNST_DL_ neurons

At the beginning of the recording sessions in current clamp mode, neurons were characterized using current pulses (450 ms in duration) from −250pA to 180pA in 10-pA increments. Based on their characteristic voltage responses,^35^ three major types of neurons were identified in the BNST_DL_. Type I neurons fire regularly and display moderate spike frequency adaptation. These neurons also display a voltage sag that indicates the presence of the hyperpolarization-activated cation current (I_h_). As a distinguishing feature of Type II neurons, post-inhibitory spikes are produced in response to preceding negative current steps that are related to the action of the low-threshold Ca^2+^ current. Additionally, Type II neurons display strong voltage sags under hyperpolarizing current pulses that indicate a high level of I_h_ current. Type III neurons differ from the previous two types in several aspects: they exhibit high rheobase and no voltage sag under negative current steps; they display prominent inward rectification that is caused by the activation of the inward rectifying K^+^ current (I_KIR_) at membrane potentials more negative than approximately −50 mV; and start firing after a characteristic slow voltage ramp mediated by the K^+^ delayed current (I_D_).

#### The effect of AVP on membrane properties and intrinsic excitability of type I, II, and III BNST_DL_ neurons

We investigated the effects of AVP and other pharmacological manipulations in Type I-III BNST_DL_ neurons on the following membrane properties: resting membrane potential (RMP), input resistance (Rin), rheobase (Rh), threshold of the first action potential (first-spike Th) (calculated as the voltage at which the depolarization rate exceeded 5 mV/ms), and latency of the first spike (first-spike Lat) evoked at the Rh current. We also investigated the intrinsic excitability by measuring the input-output (I/O) relationship before (pre), during, and after AVP application (post). We measured the steady-state frequency (SSF) by applying depolarizing pulses (1 s in duration) of different amplitudes (0–180 pA in 10 pA increments) and determining the average of the inverse of the inter-spike intervals (ISI) from all action potentials starting from the second action potential. We used SSF as a function of current to assess the effect of AVP on intrinsic excitability of Type I-III BNST_DL_ neurons from wild-type rats, as well as fluorescent BNST_DL_-OTR neurons and BNST_DL_-CRF neurons, from OTR-Cre and CRF-Cre transgenic rats, respectively.

#### Electrophysiological recordings from fluorescent neurons

To visualize OTR- and CRF-mCherry fluorescent neurons in brain slices from OTR-Cre and CRF-Cre rats or AVP-eYFP neurons in the hypothalamus (SON, SCN, and the PVN), we used an upright microscope (Scientifica Slice Scope Pro 1000 fitted with fluorescent filters (49008_Olympus BX2_Mounted, ET mCherry, Texas Red, ET560/40x ET630/75m T585lpxr for the visualization of mCherry and 49002 ET-EFP (FITC/Cy2) ET470/40x ET525/50m T495LPXR for the visualization of eYFP) and infrared differential interference contrast [IR-DIC] optics), with a CoolLED pE-300^ultra^, broad-spectrum LED illumination system as the light source. We identified fluorescent neurons using the 40x objective, mCherry or eYFP filter, and a live-image video camera (IR-2000, Dage-MTI). Once these neurons were targeted and considered healthy using an IR-DIC filter, we performed the whole-cell patch-clamp recordings.

#### Stereotaxic surgeries and adeno-associated virus (AAV) injections

We deeply anesthetized male OTR-Cre rats (*n* = 92), Wistar CRF-Cre rats (*n* = 26), both at approximately 250g of body weight, +/−10 g), and AVP-Cre rats (*n* = 83, at 220g (+/−10 g) body weight) with a mix of isoflurane (2%) and oxygen, placed them in a stereotaxic frame (Model 955, Kopf, CA) as before,^26,103^ and subcutaneously injected ketoprofen (5 mg/kg; Zoetis) for analgesia. After cleaning the rat’s head with iodide solution and applying one or two drops of lidocaine (2%, Wockhardt USA, LLC, NDC60432–464-00) to anesthetize the skin, we made a small incision to gently lift the skin and expose the skull. After making two small bilateral holes into the skulls of OTR-Cre and CRF-Cre rats, we bilaterally injected a Cre-dependent adeno-associated viral vector pAAV-hSyn-DIO-hM4D(Gi)-mCherry, which was a gift from Bryan Roth (Addgene plasmid #44362; http://n2t.net/addgene:44362; RRID:Addgene_44362)^104^ into the BNST_DL_ (at the following coordinates from bregma: 15° coronal angle, AP: 0.0 mm, ML: ±3.4 mm, DV: −7.1 mm, 100 nL) using a 5-μL Hamilton syringe (Hamilton Co., Reno, Nevada) at a rate of 25 nL/min. In a separate group of male AVP-Cre rats, we injected pAAV-hSyn FLEx-mGFP-2A-synaptophysin-mRuby (gifted by Liqun Luo (Addgene plasmid #71760; http://n2t.net/addgene:71760; RRID:Addgene_71760) bilaterally into the SON (*n* = 4, from bregma: AP −1.44, ML +/−2.07, DV −9.047, angle 0°) or SCN (*n* = 4, AP −0.58, ML -+2.015, DV −9.1, angle 12.3°). For optogenetic experiments, we injected the SON (*n* = 28), SCN (*n* = 30), or PVN (*n* = 10, coordinates from bregma: AP −1.4, ML +/−0.6 DV −7.8 angle 0°) of another cohort of male AVP-Cre rats with a Cre-dependent AAV driving channelrhodopsin (ChR2) expression pAAV-EF1a-double floxed-hChR2(H134R)-EYFP-WPRE-HGHpA (gifted by Karl Deisseroth; Addgene plasmid #20298; http://n2t.net/addgene:20298; RRID:Addgene_20298; 100 nL, diluted 1:4 with sterile saline). A separate cohort of SD rats was injected bilaterally in the PVN (*n* = 10) with the AAV driving ChR2-mCherry expression under the OT promoter (AAV-OTp-ChR2-mCherry, developed by Valery Grinevich^32^). Coordinates were based on the rat brain atlas^105^ and our prior work^26,29,32^ and adjusted based on body weight, when needed, according to the published formula.^106^ Syringes were left in place for 8 min after viral infusion before syringe retraction. Upon completion of surgery, we sutured the skin and applied antibiotic ointment to the incision site. Rats received another injection of ketoprofen 24-h post-surgery and were housed for another three weeks before experiments began. Fluorescence expression in brain sections from all injected transgenic rats was analyzed prior to inclusion of the animal in the analysis for electrophysiology, neuronal tract tracing, or behavioral experiments.

#### BNST cannulations

Stereotaxic surgeries and guide cannula implantations (22-gauge, 7 mm length; Plastics One, Roanoke, VA) for intra-BNST infusions of vasopressin or vehicle were performed in Sprague-Dawley rats as previously described.^26^ Cannulas were bilaterally implanted 2 mm above the BNST_DL_ at the following coordinates: AP: 0.0 mm, ML: ±3.4 mm, DV: −5.1 mm (for 2 mm injectors). They were secured to the skull using three stainless steel screws and dental cement. Following implantation, rats were singly housed for 24 h before being returned to social housing. Rats were allowed 4–5 days of recovery, during which they were handled daily and their cannulas checked to habituate them to the injection procedure. A dummy cannula (7 mm length; Plastics One, Roanoke, VA) was inserted between experiments to prevent obstructions. Cannula placement was determined with 1% Chicago Blue dye’s injections (Figure S1).

#### Neuronal tract tracing, dual-immunofluorescence, and microscopy

Three weeks after surgeries, rats were either used for electrophysiology as above or were perfused for neuronal tract tracing. Following the electrophysiological recordings, all slices were collected, processed, and analyzed for fluorescence in the injection sites and fibers in the BNST_DL_. In the latter case, rats received an i.p. injection of euthanasia III (Covetrus, Columbus, OH) before transcardial perfusion with cold PBS (0.05 M, pH = 7.4), followed by stabilized 10% formalin solution (200–250 mL, Fisher Scientific, SF98–4). Brains were dissected, stored in 10% formalin for an hour at room temperature, and then incubated in 30% sucrose-PBS for 48 h at 4°C. Frontal 50-μm-thick serial brain sections containing the BNST and the hypothalamus were sliced using a freezing-stage Microtome (model SM2000R, Leica Biosystems, Nussloch, Germany) and processed for immunofluorescence. Brain sections from AVP-Cre rats injected with a Cre-dependent-synaptophysin-mRuby into the SON or SCN for neuronal tract tracing and from AVP-Cre rats injected with a Cre-dependent-ChR2-eYFP (post-electrophysiology) were processed with AVP antibody (dilution 1:7500, rabbit, Millipore Sigma AB-1565, RRID:AB_90782) as before^29^ or AVP antibody generated and kindly provided by Dr. Harold Gainer (dilution 1:1000, mouse, NIH; Bethesda; USA),^107^ and then visualized with goat anti-rabbit IgG Alexa 647 secondary antibody (dilution 1:500, Invitrogen A21245, RRID:AB_2535813) or Alexa 594 secondary antibody (dilution 1:500, Invitrogen A11037, RRID:AB_2534095), respectively. These sections were then mounted with Mowiol antifade reagent on gelatin-subbed slides (Fischer Scientific, 12–544-7). We first imaged mRuby or EYFP in AVP cell bodies in the SON or SCN and fibers in the BNST with a Nikon Eclipse fluorescent microscope and then used an Olympus FV10i confocal microscope (Fluoview FV10i confocal laser-scanning microscope, Olympus, Waltham, MA, RRID:SCR_014215) to take high-magnification images and Z-stacks to visualize hypothalamic cell bodies and fibers in the BNST co-expressing mRuby or EYFP and AVP peptide.

To control for potential signal photo-bleeding between mRuby and vasopressin visualized with Alexa 647, we imaged mRuby expression without AVP immunolabeling in a subset of hypothalamic and BNST sections from the AVP-Cre injected rats, using Nikon (10x) and confocal Olympus (60x) microscopes. To determine AVP fiber expression in the BNST, brain slices from male Sprague-Dawley rats were processed for AVP immunofluorescence using the mouse AVP antibody from Dr. Gainer (NIH),^107^ visualized with Alexa Fluor 488 (RRID:AB_2534088), as described above.

The expression and distribution of OTR neurons in the BNST were determined on the brain sections from OTR-Cre rats (*n* = 9) injected with a Cre-dependent AAV driving Gi-DREADD-mCherry into the BNST_DL_. To determine the phenotype of OTR neurons, we used BNST sections from these rats combined with antibodies against two enzymes involved in cellular signaling in the BNST,^33,108^ which mark mutually exclusive neuronal populations in the BNST^70^: striatal-enriched protein tyrosine phosphatase (STEP), using anti-STEP primary antibody (mouse, dilution 1:500, Santa Cruz sc-23892, RRID:AB_2173549)^33^ and protein kinase C delta (PKCδ), with anti-PKCδ primary antibody (mouse, dilution 1:1000, BD Biosciences 610398, RRID:AB_397781).^108^ Both proteins were then visualized with goat anti-mouse IgG Alexa 488 secondary antibody (dilution 1:500, Invitrogen A11029, RRID:AB_2534088). Confocal microscopy was used for high-resolution images and to acquire multi-Z-stack images at 60x for cell quantification.

To count OTR-, STEP-, and PKCδ-expressing neurons and neurons co-expressing OTR and STEP or OTR and PKCδ, we took z-stacks from the entire dorsal BNST (above the anterior commissure) from 3 rats, selecting the Multi Area Z-stacks Time Lapse function in the confocal Olympus Fv10i Fluoview software (RRID:SCR_014215). Each z stack contained 25–35 confocal planes with 1-μm intervals with 60x magnification. Z-stacks were taken at anterior (bregma −0.12 mm), middle (bregma −0.24 mm), and posterior (bregma −0.48 mm) regions of the antero-posterior axis of the BNST_DL_. OTR-, STEP-, and PKCδ-expressing neurons were counted on the left and right hemispheres from 3 sections per rat and averaged per BNST. After image acquisition, all individual z-stacks were used to create a montage of the entire dorsal BNST using the function Multi Stack Montage, which is part of the BIOP plugin package on the ImageJ software (ImageJ, NIH, RRID: SCR_003070, Image processing and analysis in Java,^109^). The final montage was used to manually count each cell type using ImageJ’s cell counter, which allows to assign a number to each cell type and label them to prevent counting the same cell twice. OTR-mCherry and STEP or PKCδ co-expressing neurons were assigned three numbers, one for OTR-mCherry, one for STEP or PKCδ, and another to indicate double labeling. Every third consecutive BNST section (150 μm intervals) from the entire brain was used for quantification.

Diagrams and graphical abstract were created and labeled using SciDraw (images/icons created by Dr. Zhe Chen, Dr. Christophe Leterrie), Biorender (Biorender.com, RRID:SCR_018361), Adobe Illustrator ((RRID:SCR_010279), Adobe Photoshop (RRID: SCR_014199), and Microsoft PowerPoint software.

#### Optogenetic stimulation protocol in AVP-Cre transgenic rats

As we previously reported detailed cellular effects of exogenous OT application in the BNST_DL_,^38^ we first validated the optogenetic approach in the rats injected with the AAV-OTp-ChR2-mCherry virus, generated by the Grinevich Lab^32^ in the PVN and recorded from brain slices containing the BNST. Tetanic blue light stimulation (TLS, 10-ms single pulse at 30Hz for 20 s, which previously successfully triggered axonal OT release in the central amygdala^32^) evoked OT release in the BNST_DL_ (Figure S2). Three weeks after Cre-dependent AAV-ChR2-eYFP was injected in the SON, PVN, or SCN, we decapitated AVP-Cre rats as above and used brain slices containing the BNST or hypothalamus for electrophysiological recordings combined with optogenetic stimulation. The slices were kept in aCSF in the dark to avoid photobleaching of the fluorescent signal and ChR2 activation. Because high frequencies of action potentials are thought to be necessary to trigger neuropeptide release, we first performed current-clamp recordings from fluorescent AVP neurons in the SON and demonstrated that 10-ms blue light pulses (BL, 470 nm) evoked action potentials in AVP SON neurons that followed frequencies up to 30Hz applied for 20 s (Figure 6E). Then, to study the effects of AVP released from terminals containing ChR2 in the BNST on the intrinsic excitability of Type I and Type III BNST_DL_ neurons, we determined the input/output (I/O) relationship in response to 80, 100, and 120pA current injections, before and after TLS. Here, we modified the optogenetic stimulation protocol to more closely mimic the physiological firing of AVP neurons in the SCN and SON^110,111^ with 10-ms single pulses at 10Hz for 20 s. This TLS protocol was used in BNST slices from AVP-Cre rats injected with Cre-dependent AAV-ChR2 in the hypothalamus and was delivered at the recording site using whole-field illumination through a 40x water-immersion objective (Olympus, Tokyo, Japan) with a pE-300^ultra^ CoolLED illumination system (CoolLED Ltd., Andover, UK). As the TLS of AVP-PVN neurons input did not produce a consistent excitatory effect in the BNST, we modified the protocol to better mimic the firing frequencies of AVP_**PVN**_ neurons. AVP neurons can fire in three modes: slow irregular (<1Hz), fast continuous (>1Hz), and phasic bursting. The latter two modes, especially phasic bursting, are associated with AVP release in response to stimuli such as dehydration and hypovolemia.^46,48^ Unlike OT neurons, this bursting is not synchronized among cells and consists of alternating periods of activity (5–15Hz) and silence, each lasting 20–40 s.^45,47^ Here, we used four bursts of blue light (at 70% maximum intensity) delivered every 30 s. Each burst lasted 15 s and consisted of 6.3Hz pulses (i.e., 94 pulses per burst), with each pulse lasting 10 msec. Finally, in a subset of neurons in each experiment, to confirm peptide receptor-mediated cellular effects, we applied the OTR antagonist, OTA (as above), before TLS.

#### Acoustic startle response (ASR) and fear-potentiated startle (FPS) in OTR-Cre rats

OTR-Cre rats (*n* = 45) injected with a Cre-dependent AAV-Gi-DREADD-mCherry were used for chemogenetic inhibition of OTR neurons in the BNST_DL_ before fear conditioning (followed by FPS) and before EPM experiments (*n* = 22 saline, *n* = 21 CNO). Only rats with robust Gi-DREADD-mCherry expression in the BNST in one or both hemispheres, or at least moderate bilateral expression, were included in the data analysis (*n* = 43).

Sprague-Dawley rats (*n* = 27) implanted with guide cannulas into the BNST_DL_ were used for AVP (10 ng/0.5μL per hemisphere) vs. vehicle (saline) infusion into the BNST_DL_, 10 min before the fear-conditioning, and again before the EPM experiments, as above. Two rats were eliminated based on the incorrect cannula placement for a total 25 rats (*n* = 12 saline, *n* = 13 AVP).

Rats were tested in Plexiglas enclosures inside sound-attenuating chambers (San Diego Instruments, Inc., CA), as described before.^26,37^ The Plexiglas enclosures were installed on top of a platform that detected movement (jump amplitude measured within a 200-ms window following the onset of the startle-eliciting noise) and transformed the velocity of the movement into a voltage output^112^ detected by SR-Lab software (Part No: 6300–0000-Q, San Diego Instruments, Inc., CA). ASR was measured during 30 trials of startle-eliciting 95-dB white noise burst (WNB) on day 1 (chamber and startle habituation) and day 2 (baseline pre-shock test). On day 3 (fear conditioning), rats received an i.p. injection of CNO (2 mg/kg) 45 min before exposure to 10 presentations of 3.7-s cue light (conditioned stimulus, CS), each co-terminating with a 0.5-s foot shock (unconditioned stimulus, US; 0.5 mA) in context A. On day 4, rats were tested for recall of cued and non-cued fear in context B, where ASR was first measured alone (10 trials, post-shock), followed by an additional 20 trials in which ASR was measured in the presence of the cue (light+noise trials) or in the absence of the cue (noise-only trials), presented in a pseudorandom order (Figures 4A and S3). Selected environmental cues were altered in context B to distinguish it from context A: context B lacked the steel grid bars used for conditioning in context A, was cleaned with a different disinfectant (ethanol 70% instead of peroxide), and had a different experimenter performing the testing. On day 5, rats were tested for contextual fear recall, where ASR was measured with no cue presentation in the original context A. To determine the rate of fear extinction, we tested rats for cued/non-cued fear three times and contextual fear two times on alternate days, see Figure S3 for FPS components. The data was acquired and collected using SR-LAB software (San Diego Instruments, CA).

#### Elevated plus maze (EPM) in OTR-Cre rats

The behavioral experiments took place in a room under a dim lighting condition. The experimental OTR-Cre rats used for the FPS test were tested on the EPM 3 days after the last FPS test. The EPM apparatus was elevated 32 inches (in) from the ground and consisted of two closed-arms (width, length, height: 5 in × 20 in × 18 in), two open-arms (width and length: 5 in × 20 in), and a squared center (width and length: 5 in × 5 in). Forty-five min after saline/CNO i.p. or 10 min after intra-BNST saline/AVP injections, rats were placed at the center of the EPM and allowed them to explore for 5 min. We measured total entries and time spent in the open-arms, closed-arms, and at the center using ANY-Maze 6.34 software (Stoelting, Wood Dale, IL, RRID:SCR_014289) and quantified time spent freezing in each of these compartments.

#### Functional verification of Gi-DREADD with *in vitro* whole-cell patch-clamp electrophysiology

The whole-cell patch recordings were performed as above^38^ on 300-μm-thick brain slices containing the BNST from 5 AAV-injected OTR-Cre male rats. To confirm that OTR-BNST_DL_ neurons expressing Gi-DREADD-mCherry were functionally inhibited by CNO, the neurons were depolarized to action potential threshold and firing activity was recorded for 5 min before bath application of CNO (20 μM), for 5 min during CNO application, and for at least 8 min during washout (Figure 4B).

#### Breeding protocols and genotyping

Rats were bred at 11–15 weeks of age by pairing Cre+/− male or female rats with wild-type SD (for OTR-Cre and AVP-Cre) or Wistar (for CRF-Cre) rats, such that all offspring were heterozygous for Cre. Wild-type breeders were purchased from Envigo, as above, from diverse litters to keep the Cre lines outbred. Pairs were together for approximately 10–14 days or until females showed signs of pregnancy and then individually housed. Rats were genotyped at weaning (postnatal day 21) for the presence of Cre. During weaning, males and females were separated and ear tagged (Braintree Scientific, Inc. 1005–1LZ). For tagging, we lightly exposed rats to the inhalant isoflurane and used an ear punch (Braintree Scientific, Inc. EP-S-902) to create a 2-mm hole for tag insertion. Ear tissue was stored in 0.2-mL 8-strip PCR tubes (GeneMate, VWR, 490003–710) labeled with the animals corresponding ID number at −20°C.

For genotyping, we added 50 μL of a lysis buffer (2.5 mL 1M Tris, pH 8.8, 100 μL 0.5M EDTApH8.0, 250ul Tween 20, 1.0 mL) with proteinase K (20 mg added to 1.0 mL 50 nM Tris-HCLpH8.0.10mM CaCl_2_ 15 μL) to each tube of tissue and placed tubes in a Thermo Cycler at 95°C overnight. The next day, we vortexed the tubes slightly and returned them to the Thermo Cycler for another 10 min at 100°C to denature the proteinase K. We then centrifuged the samples for 10 min at 6000 rpm and collected the supernatant for use with the PCR mix. The primers used were specific for the Cre-sequence within the DNA, amplifying only the DNA of Cre+ animals with the following sequence (5’ → 3′) for OTR-Cre and AVP-Cre: Cre 3 (forward) TCGCTGCATTACCGGTCGATGC (22 bp), and Cre 4 (reverse) CCATGAGTGAACGAACCTGGTCG (23 bp), and for CRF-Cre: SG13 (forward) GCATTACCGGTCGATGCAACGAGTGATGAG, and SG14 (reverse) GAGTGAACGAACCTGGTCGAAATCAGTGCG (all from ThermoFisher Scientific), as before.^39^ The PCR-mix was composed of the extracted DNA, primers, KAPA2G Fast HS Master Mix (Kapa Biosystems, Inc, KK5621), Ultrapure BSA (Invitrogen, AM2616) and nuclease free H_2_O. The PCR-Mix and protocol used for the thermal cycler is below. The PCR samples were loaded on a 2.0% agarose gel (VWR, 0710) made in 1X TAE buffer (VWR, 82021–492) with 50 μL ethidium bromide (0.625 mg/mL, VWR, E406) in an electrophoresis chamber containing 1X TAE buffer at 80 V for 90 min. A 10-Kb DNA ladder (BioLabs, Inc., N3270S) was run in parallel to verify the size of the amplified DNA fragment.

### QUANTIFICATION AND STATISTICAL ANALYSIS

Electrophysiological data are presented as mean ± standard error of mean (SEM). The distribution of datasets was first evaluated with Kolmogorov-Smirnov test. When normal distribution was observed, the effects of AVP and other drugs on membrane properties were analyzed by a repeated measures (RM) one-way analysis of variance (ANOVA) or mixed-effects model, when applicable. The effects of AVP (and other pharmacological manipulations) on intrinsic excitability and firing frequency were analyzed with RM two-way ANOVA with treatment and current as factors. Where the F-ratio was significant, all-pairwise post hoc comparisons were made using Tukey’s or Sidak’s tests.

Behavioral data are presented as violin plots depicting the median and the 25^th^ and 75^th^ quartiles. FPS data were analyzed by a two-way RM ANOVA with the factors trial type (pre-shock, post-shock, noise-only, light+noise) and treatment (saline vs. CNO). When F-ratio was significant, all *post hoc* analyses were compared using Sidak’s multiple comparison test. For analyses of the effect of treatment on cued, non-cued, and contextual fear, data are presented as percentage change scores of ASR and were analyzed with un-paired *t*-tests. The percentage change of ASR was analyzed according to the following formulas:

Cuedfear=(LightNoise−Noisealone)/Noisealone)×100%incontextB

Non−cuedfear=(Noisealone−Postshock)/Postshock)×100%incontextB

Contextualfear=(Postshock−Preshock)/Preshock)×100%incontextA

Shock reactivity for each individual rat was calculated as the average startle amplitude during each of the 10 foot-shock presentations during fear conditioning (no WNB present) and was analyzed with un-paired *t*-tests, See Figure S3 for the FPS components.

EPM data are presented as the total number of entries and percentage of time spent in each compartment. Total time freezing in each compartment is expressed in seconds. Comparisons between saline and CNO groups were analyzed for each parameter with an un-paired *t* test.

All statistical analyses were completed using GraphPad Prism version 10.2.1 (339) (GraphPad Software, Inc., San Diego, CA, RRID: SCR_002798). *p < 0.05* was considered significant.

The statistical details of the experiments are provided in the Results section and in Figure Legends. In electrophysiological experiments, n represents the number of neurons recorded per number of rats used per group (both are reported), whereas in behavioral experiments n denotes a number of rats per group.

## Supplementary Material

1

Supplemental information can be found online at https://doi.org/10.1016/j.celrep.2025.115768.

## Figures and Tables

**KEY RESOURCES TABLE:** 

REAGENT or RESOURCE	SOURCE	IDENTIFIER

Antibodies		

Mouse anti-arginine-vasopressin (AVP) antibody	Dr. Harold Gainer (NIH, Bethesda, USA)	NA
Rabbit AVP antibody	Millipore-Sigma, AB-1565	RRID:AB_90782
Mouse oxytocin (OT) antibody	Millipore-Sigma, MAB5296	RRID:AB_11212999
Mouse Striatal enriched protein tyrosine phosphatase (STEP) antibody	Santa Cruz Antibodies, sc-23892	RRID:AB_2173549
Mouse Protein kinase C delta (PKCδ) primary antibody	BD Biosciences, 610398	RRID:AB_397781
Goat anti-rabbit IgG Alexa 488 secondary antibody	ThermoFisher Scientific, A11034	RRID:AB_2576217
Goat anti-rabbit IgG Alexa 594 secondary antibody	ThermoFisher Scientific, A11037	RRID:AB_2534095
Goat anti-rabbit IgG Alexa 647 secondary antibody	ThermoFisher Scientific, A21245	RRID:AB_2535813
Goat anti-mouse IgG Alexa 488 secondary antibody	ThermoFisher Scientific, A11029	RRID:AB_2534088
Goat anti-mouse IgG Alexa 594 secondary antibody	ThermoFisher Scientific, A32742	RRID:AB_2762825
Goat anti-mouse IgG Alexa 647 secondary antibody	ThermoFisher Scientific, A21236	RRID:AB_2535805
Streptavidin-Alexa Fluor 594 conjugate	ThermoFisher Scientific, S32356	
Streptavidin-Alexa Fluor 488 conjugate	ThermoFisher Scientific, S11223	

Chemicals, peptides, and recombinant proteins		

Arginine-Vasopressin (AVP)	Bachem Americas	4012215
Oxytocin (OT)	Bachem Americas	4016373
([Thr,^4^Gly^7^]-oxytocin, OTR agonist (TGOT)	Bachem Americas	4013837
V1aR agonist (FE201874)	Ferring Pharmaceuticals, Inc.	N/A
V1bR agonist (d[Cha4]-AVP)	GlpBio Technology, Inc.	GC1659
V1aR/OTR antagonist (d(CH_2_)_5_[Tyr(Me)^2^] AVP) (Manning compound)	Tocris, Bio-Techne Corporation	3377
OTR antagonist (OTA), d(CH2)5(1), D-Tyr(2), Thr(4), Orn(8), des-Gly-NH2(9)]-Vasotocin trifluoroacetate salt	Chemical Repository, NIMH	V-905
V1aR antagonist (SR49059)	Tocris, Bio-Techne Corporation	2310
V1bR antagonist (Nelivaptan)	Tocris, Bio-Techne Corporation	6195
AMPA/kainate receptor antagonist (CNQX)	Tocris, Bio-Techne Corporation	1045
NMDA receptor antagonist (D-AP5)	Tocris, Bio-Techne Corporation	0106
GABA-A receptor antagonist (Picrotoxin, PTX)	Tocris, Bio-Techne Corporation	1128
DREADDs ligand (Clozapine-N-oxide, CNO)	Tocris, Bio-Techne Corporation	4936
Euthanasia III	(Covetrus, Columbus, OH)	080661
Neurobiotin	Vector Laboratories, Burlingame, CA	SP-1120

Deposited data		

Raw and searchable research data in neuroscience and physiology	ndi-cloud.com	https://www.ndi-cloud.com/datasets/67f723d574f5f79c6062389d; accession number: 67f723d574f5f79c6062389d; https://doi.org/10.63884/ndic.2025.jyxfer8m; ndi-cloud.com
Custom MATLAB scripts for data acquisition and analysis	Zenodo.org	https://doi.org/10.5281/zenodo.15238413

Experimental models: Organisms/strains		

Sprague-Dawley rats, male	Envigo, Chicago, IL	RRID:MGI:5651135
CRF-Cre transgenic rats	Dr. Robert Messing, University of Texas Austin	NA
OTR-Cre transgenic rats	Dr. Valery Grinevich, Heidelberg University, Germany	NA
AVP-Cre transgenic rats	Dr. Valery Grinevich, Heidelberg University, Germany	NA

Oligonucleotides		

OTR-Cre Forward (Cre 3)	Thermo Fisher Scientific	TCGCTGCATTACCGGTCGATGC
OTR-Cre Reverse (Cre 4)	Thermo Fisher Scientific	CCATGAGTGAACGAACCTGGTCG
AVP-Cre Forward (Cre 3)	Thermo Fisher Scientific	TCGCTGCATTACCGGTCGATGC
AVP-Cre Reverse (Cre 4)	Thermo Fisher Scientific	CCATGAGTGAACGAACCTGGTCG
CRF-Cre Forward (SG13)	Thermo Fisher Scientific	GCATTACCGGTCGATGCAACGAGTGATGAG
CRF-Cre Reverse (SG14)	Thermo Fisher Scientific	GAGTGAACGAACCTGGTCGAAATCAGTGCG

Recombinant DNA		

pAAV-hSyn-FLEx-mGFP-2A-synaptophysin-mRuby	Addgene	RRID:Addgene_71760
pAAV8-hSyn-DIO-hM4D(Gi)-mCherry	Addgene	RRID:Addgene_44362
pAAV1-EF1a-double-floxed-hChR2(H134R)-eYFP-WPRE-HGHpA	Addgene	RRID:Addgene_20298
AAV-OTp-ChR2-mCherry	Dr. Valery Grinevich, Heidelberg University, Germany	N/A

Software and algorithms		

Prism, Version 10.0, Statistical analysis software	GraphPad	RRID:SCR_002798
Electrophysiology data acquisition, Axon pCLAMP 11	Molecular Devices	RRID:SCR_011323
MATLAB	Math-Work, Natick, MA	RRID:SCR_001622
SR-LAB, startle acquisition software	San Diego Instruments, CA	
ANY-Maze (6.34, behavioral software)	Stoelting, Wood Dale, IL	RRID:SCR_014289
Imaging software, ImageJ	NIH, Bethesda	RRID:SCR_003070
Figure preparation software	Biorender	RRID:SCR_018361
Olympus Fluoview Fv10i software	Olympus	RRID:SCR_014215
Adobe Illustrator	Adobe	RRID:SCR_010279
Adobe Photoshop	Adobe	RRID:SCR_014199
SciDraw, Imaging Software	(images/icons created by Dr. Zhe Chen, Dr. Christophe Leterrie)	

## Highlights

- Vasopressin excites neurons of the BNST via oxytocin receptor (OTR)
- Vasopressin excites OTR- and CRF-expressing neurons of the BNST
- The BNST receives functional vasopressinergic inputs from various hypothalamic nuclei
- OTR-expressing BNST neurons play an anxiolytic function in male rats

## References

[1] Kalsbeek A, Fliers E, Hofman MA, Swaab DF, and Buijs RM (2010). Vasopressin and the output of the hypothalamic biological clock. J. Neuroendocrinol. 22, 362–372. 20088910 10.1111/j.1365-2826.2010.01956.x

[2] Brown CH, Ludwig M, Tasker JG, and Stern JE (2020). Somatodendritic vasopressin and oxytocin secretion in endocrine and autonomic regulation. J. Neuroendocrinol. 32, e12856. 32406599 10.1111/jne.12856 PMC9134751

[3] Serradeil-Le Gal C, Wagnon J 3rd, Tonnerre B, Roux R, Garcia G, Griebel G, and Aulombard A (2005). An overview of SSR149415, a selective nonpeptide vasopressin V(1b) receptor antagonist for the treatment of stress-related disorders. CNS Drug Rev. 11, 53–68. 15867952 10.1111/j.1527-3458.2005.tb00035.x PMC6741711

[4] Caldwell HK, Lee H-J, Macbeth AH, and Young WS (2008). Vasopressin: behavioral roles of an ‘original’ neuropeptide. Prog. Neurobiol. 84, 1–24. 18053631 10.1016/j.pneurobio.2007.10.007 PMC2292122

[5] Veenema AH (2012). Toward understanding how early-life social experiences alter oxytocin- and vasopressin-regulated social behaviors. Horm. Behav. 61, 304–312. 22197269 10.1016/j.yhbeh.2011.12.002

[6] Jurek B, and Neumann ID (2018). The Oxytocin Receptor: From Intracellular Signaling to Behavior. Physiol. Rev. 98, 1805–1908. 29897293 10.1152/physrev.00031.2017

[7] Janeček M, and Dabrowska J (2019). Oxytocin facilitates adaptive fear and attenuates anxiety responses in animal models and human studies—potential interaction with the corticotropin-releasing factor (CRF) system in the bed nucleus of the stria terminalis (BNST). Cell Tissue Res. 375, 143–172. 30054732 10.1007/s00441-018-2889-8 PMC6336503

[8] Olivera-Pasilio V, and Dabrowska J (2020). Oxytocin Promotes Accurate Fear Discrimination and Adaptive Defensive Behaviors. Front. Neurosci. 14, 583878. 33071751 10.3389/fnins.2020.583878 PMC7538630

[9] Khan S, Raghuram V, Chen L, Chou CL, Yang CR, Khundmiri SJ, and Knepper MA (2024). Vasopressin V2 receptor, tolvaptan, and ERK1/2 phosphorylation in the renal collecting duct. Am. J. Physiol. Ren. Physiol. 326, F57–F68. 10.1152/ajprenal.00124.2023 PMC10812694 37916285

[10] Lolait SJ, O’Carroll AM, Mahan LC, Felder CC, Button DC, Young WS 3rd, Mezey E, and Brownstein MJ (1995). Extrapituitary expression of the rat V1b vasopressin receptor gene. Proc. Natl. Acad. Sci. USA 92, 6783–6787. 7624319 10.1073/pnas.92.15.6783 PMC41413

[11] Corbani M, Marir R, Trueba M, Chafai M, Vincent A, Borie AM, Desarménien MG, Ueta Y, Tomboly C, Olma A, (2018). Neuroanatomical distribution and function of the vasopressin V1B receptor in the rat brain deciphered using specific fluorescent ligands. Gen. Comp. Endocrinol. 258, 15–32. 29155265 10.1016/j.ygcen.2017.10.011

[12] Tribollet E, Barberis C, Jard S, Dubois-Dauphin M, and Dreifuss JJ (1988). Localization and pharmacological characterization of high affinity binding sites for vasopressin and oxytocin in the rat brain by light microscopic autoradiography. Brain Res. 442, 105–118. 2834008 10.1016/0006-8993(88)91437-0

[13] Tribollet E, Dubois-Dauphin M, Dreifuss JJ, Barberis C, and Jard S (1992). Oxytocin receptors in the central nervous system. Distribution, development, and species differences. Ann. N. Y. Acad. Sci. 652, 29–38. 1320828 10.1111/j.1749-6632.1992.tb34343.x

[14] Johnson AE, Audigier S, Rossi F, Jard S, Tribollet E, and Barberis C (1993). Localization and characterization of vasopressin binding sites in the rat brain using an iodinated linear AVP antagonist. Brain Res. 622, 9–16. 8242389 10.1016/0006-8993(93)90795-o

[15] Roozendaal B, Schoorlemmer GH, Wiersma A, Sluyter S, Driscoll P, Koolhaas JM, and Bohus B (1992). Opposite effects of central amygdaloid vasopressin and oxytocin on the regulation of conditioned stress responses in male rats. Ann. N. Y. Acad. Sci. 652, 460–461. 1626849 10.1111/j.1749-6632.1992.tb34384.x

[16] Viviani D, and Stoop R (2008). Opposite effects of oxytocin and vasopressin on the emotional expression of the fear response. Prog. Brain Res. 170, 207–218. 18655884 10.1016/S0079-6123(08)00418-4

[17] Veinante P, and Freund-Mercier MJ (1997). Distribution of oxytocin- and vasopressin-binding sites in the rat extended amygdala: a histoautoradiographic study. J. Comp. Neurol. 383, 305–325. 9205043

[18] Smith CJW, DiBenedictis BT, and Veenema AH (2019). Comparing vasopressin and oxytocin fiber and receptor density patterns in the social behavior neural network: Implications for cross-system signaling. Front. Neuroendocrinol. 53, 100737. 30753840 10.1016/j.yfrne.2019.02.001 PMC7469073

[19] Bayerl DS, Hönig JN, and Bosch OJ (2016). Vasopressin V1a, but not V1b, receptors within the PVN of lactating rats mediate maternal care and anxiety-related behaviour. Behav. Brain Res. 305, 18–22. 26909846 10.1016/j.bbr.2016.02.020

[20] Hernández-Pérez OR, Crespo-Ramírez M, Cuza-Ferrer Y, Anias-Calderón J, Zhang L, Roldan-Roldan G, Aguilar-Roblero R, Borroto-Escuela DO, Fuxe K, and Perez de la Mora M (2018). Differential activation of arginine-vasopressin receptor subtypes in the amygdaloid modulation of anxiety in the rat by arginine-vasopressin. Psychopharmacology 235, 1015–1027. 29306965 10.1007/s00213-017-4817-0

[21] Rood BD, Stott RT, You S, Smith CJW, Woodbury ME, and De Vries GJ (2013). Site of origin of and sex differences in the vasopressin innervation of the mouse (Mus musculus) brain. J. Comp. Neurol. 521, 2321–2358. 23239101 10.1002/cne.23288

[22] Rigney N, de Vries GJ, and Petrulis A (2023). Sex differences in afferents and efferents of vasopressin neurons of the bed nucleus of the stria terminalis and medial amygdala in mice. Horm. Behav. 154, 105407. 37523807 10.1016/j.yhbeh.2023.105407 PMC10529859

[23] Mohr E, Bahnsen U, Kiessling C, and Richter D (1988). Expression of the vasopressin and oxytocin genes in rats occurs in mutually exclusive sets of hypothalamic neurons. FEBS Lett. 242, 144–148. 3203740 10.1016/0014-5793(88)81003-2

[24] Sullivan GM, Apergis J, Bush DEA, Johnson LR, Hou M, and Ledoux JE (2004). Lesions in the bed nucleus of the stria terminalis disrupt corticosterone and freezing responses elicited by a contextual but not by a specific cue-conditioned fear stimulus. Neuroscience 128, 7–14. 15450349 10.1016/j.neuroscience.2004.06.015

[25] Duvarci S, Bauer EP, and Pare D (2009). The Bed Nucleus of the Stria Terminalis Mediates Inter-individual Variations in Anxiety and Fear. J. Neurosci. 29, 10357–10361. 19692610 10.1523/JNEUROSCI.2119-09.2009 PMC2741739

[26] Moaddab M, and Dabrowska J (2017). Oxytocin receptor neurotransmission in the dorsolateral bed nucleus of the stria terminalis facilitates the acquisition of cued fear in the fear-potentiated startle paradigm in rats. Neuropharmacology 121, 130–139. 28456687 10.1016/j.neuropharm.2017.04.039 PMC5553312

[27] Goode TD, Ressler RL, Acca GM, Miles OW, and Maren S (2019). Bed nucleus of the stria terminalis regulates fear to unpredictable threat signals. Elife 8, e46525. 30946011 10.7554/eLife.46525 PMC6456295

[28] DiBenedictis BT, Nussbaum ER, Cheung HK, and Veenema AH (2017). Quantitative mapping reveals age and sex differences in vasopressin, but not oxytocin, immunoreactivity in the rat social behavior neural network. J. Comp. Neurol. 525, 2549–2570. 28340511 10.1002/cne.24216 PMC6066795

[29] Dabrowska J, Hazra R, Ahern TH, Guo JD, McDonald AJ, Mascagni F, Muller JF, Young LJ, and Rainnie DG (2011). Neuroanatomical evidence for reciprocal regulation of the corticotrophin-releasing factor and oxytocin systems in the hypothalamus and the bed nucleus of the stria terminalis of the rat: Implications for balancing stress and affect. Psychoneuroendocrinology 36, 1312–1326. 21481539 10.1016/j.psyneuen.2011.03.003 PMC3142325

[30] Duque-Wilckens N, Steinman MQ, Laredo SA, Hao R, Perkeybile AM, Bales KL, and Trainor BC (2016). Inhibition of vasopressin V1a receptors in the medioventral bed nucleus of the stria terminalis has sex- and context-specific anxiogenic effects. Neuropharmacology 110, 59–68. 27452721 10.1016/j.neuropharm.2016.07.018 PMC5028294

[31] Luo PX, Zakharenkov HC, Torres LY, Rios RA, Gegenhuber B, Black AM, Xu CK, Minie VA, Tran AM, Tollkuhn J, and Trainor BC (2022). Oxytocin receptor behavioral effects and cell types in the bed nucleus of the stria terminalis. Horm. Behav. 143, 105203. 35636023 10.1016/j.yhbeh.2022.105203 PMC9827713

[32] Knobloch HS, Charlet A, Hoffmann LC, Eliava M, Khrulev S, Cetin AH, Osten P, Schwarz MK, Seeburg PH, Stoop R, and Grinevich V (2012). Evoked axonal oxytocin release in the central amygdala attenuates fear response. Neuron 73, 553–566. 22325206 10.1016/j.neuron.2011.11.030

[33] Dabrowska J, Hazra R, Guo JD, Li C, Dewitt S, Xu J, Lombroso PJ, and Rainnie DG (2013). Striatal-Enriched Protein Tyrosine Phosphatase—STEPs Toward Understanding Chronic Stress-Induced Activation of Corticotrophin Releasing Factor Neurons in the Rat Bed Nucleus of the Stria Terminalis. Biol. Psychiatry 74, 817–826. 24012328 10.1016/j.biopsych.2013.07.032 PMC3818357

[34] Dabrowska J, Hazra R, Guo J-D, DeWitt S, and Rainnie DG (2013). Central CRF neurons are not created equal: phenotypic differences in CRF-containing neurons of the rat paraventricular hypothalamus and the bed nucleus of the stria terminalis. Front. Neurosci. 7, 156. 24009552 10.3389/fnins.2013.00156 PMC3757458

[35] Hammack SE, Mania I, and Rainnie DG (2007). Differential Expression of Intrinsic Membrane Currents in Defined Cell Types of the Anterolateral Bed Nucleus of the Stria Terminalis. J. Neurophysiol. 98, 638–656. 17537902 10.1152/jn.00382.2007

[36] Rodríguez-Sierra OE, Turesson HK, and Pare D (2013). Contrasting distribution of physiological cell types in different regions of the bed nucleus of the stria terminalis. J. Neurophysiol. 110, 2037–2049. 23926040 10.1152/jn.00408.2013 PMC3841931

[37] Martinon D, Lis P, Roman AN, Tornesi P, Applebey SV, Buechner G, Olivera V, and Dabrowska J (2019). Oxytocin receptors in the dorsolateral bed nucleus of the stria terminalis (BNST) bias fear learning toward temporally predictable cued fear. Transl. Psychiatry 9, 140. 31000694 10.1038/s41398-019-0474-x PMC6472379

[38] Francesconi W, Berton F, Olivera-Pasilio V, and Dabrowska J (2021). Oxytocin excites BNST interneurons and inhibits BNST output neurons to the central amygdala. Neuropharmacology 192, 108601. 33971215 10.1016/j.neuropharm.2021.108601 PMC8297366

[39] Althammer F, Roy RK, Lefevre A, Najjar RS, Schoenig K, Bartsch D, Eliava M, Feresin RG, Hammock EAD, Murphy AZ, (2022). Altered PVN-to-CA2 hippocampal oxytocin pathway and reduced number of oxytocin-receptor expressing astrocytes in heart failure rats. J. Neuroendocrinol. 34, e13166. 35657290 10.1111/jne.13166 PMC9495289

[40] Iwasaki M, Lefevre A, Althammer F, Clauss Creusot E, Łąpieś O, Petitjean H, Hilfiger L, Kerspern D, Melchior M, Küppers S, (2023). An analgesic pathway from parvocellular oxytocin neurons to the periaqueductal gray in rats. Nat. Commun. 14, 1066. 36828816 10.1038/s41467-023-36641-7 PMC9958129

[41] Pomrenze MB, Millan EZ, Hopf FW, Keiflin R, Maiya R, Blasio A, Dadgar J, Kharazia V, De Guglielmo G, Crawford E, (2015). A Transgenic Rat for Investigating the Anatomy and Function of Corticotrophin Releasing Factor Circuits. Front. Neurosci. 9, 487. 26733798 10.3389/fnins.2015.00487 PMC4689854

[42] Allen Institute for Brain Science. (2024). Allen Mouse Brain Atlas (2004). Retrieved from https://mouse.brain-map.org (Accessed March 30, 2024). http://atlas.brain-map.org/atlas?atlas=1#atlas=1&plate=100960312&structure=554&x=5222.879464285715&y=3571.841212681362&zoom=-3&resolution=11.97&z=6

[43] Olivera-Pasilio V, and Dabrowska J (2023). Fear-Conditioning to Unpredictable Threats Reveals Sex and Strain Differences in Rat Fear-Potentiated Startle (FPS). Neuroscience 530, 108–132. 37640137 10.1016/j.neuroscience.2023.08.030 PMC10726736

[44] Chudoba R, and Dabrowska J (2024). Corticotropin-releasing factor neurons of the bed nucleus of the stria terminalis demonstrate sex- and estrous phase-dependent differences in synaptic activity and in their role in anxiety-potentiated startle. Preprint at bioRxiv. 10.1101/2024.11.15.623898v2. PMC12442882 40738418

[45] Wakerley JB, Poulain DA, and Brown D (1978). Comparison of firing patterns in oxytocin- and vasopressin-releasing neurones during progressive dehydration. Brain Res. 148, 425–440. 656941 10.1016/0006-8993(78)90730-8

[46] Li C, Tripathi PK, and Armstrong WE (2007). Differences in spike train variability in rat vasopressin and oxytocin neurons and their relationship to synaptic activity. J. Physiol. 581, 221–240. 17332000 10.1113/jphysiol.2006.123810 PMC2075210

[47] Poulain DA, and Wakerley JB (1982). Electrophysiology of hypothalamic magnocellular neurones secreting oxytocin and vasopressin. Neuroscience 7, 773–808. 6124897 10.1016/0306-4522(82)90044-6

[48] Dutton A, and Dyball RE (1979). Phasic firing enhances vasopressin release from the rat neurohypophysis. J. Physiol. 290, 433–440. 469785 10.1113/jphysiol.1979.sp012781 PMC1278845

[49] Marir R, Virsolvy A, Wisniewski K, Mion J, Haddou D, Galibert E, Meraihi Z, Desarménien MG, and Guillon G (2013). Pharmacological characterization of FE 201874, the first selective high affinity rat V _1A_ vasopressin receptor agonist: FE 201874, a selective rat V _1A_ agonist. Br. J. Pharmacol. 170, 278–292. 23725319 10.1111/bph.12249 PMC3834753

[50] Viviani D, Charlet A, van den Burg E, Robinet C, Hurni N, Abatis M, Magara F, and Stoop R (2011). Oxytocin selectively gates fear responses through distinct outputs from the central amygdala. Science 333, 104–107. 21719680 10.1126/science.1201043

[51] Owen SF, Tuncdemir SN, Bader PL, Tirko NN, Fishell G, and Tsien RW (2013). Oxytocin enhances hippocampal spike transmission by modulating fast-spiking interneurons. Nature 500, 458–462. 23913275 10.1038/nature12330 PMC5283693

[52] Hu B, Boyle CA, and Lei S (2021). Activation of Oxytocin Receptors Excites Subicular Neurons by Multiple Signaling and Ionic Mechanisms. Cerebr. Cortex 31, 2402–2415. 10.1093/cercor/bhaa363 PMC8023860 33341872

[53] Migita K, Hori N, Manako J, Saito R, Takano Y, and Kamiya H (1998). Effects of arginine-vasopressin on neuronal interaction from the area postrema to the nucleus tractus solitarii in rat brain slices. Neurosci. Lett. 256, 45–48. 9832213 10.1016/s0304-3940(98)00753-8

[54] Boyle CA, Hu B, Quaintance KL, and Lei S (2021). Involvement of TRPC5 channels, inwardly rectifying K+ channels, PLCβ and PIP2 in vasopressin-mediated excitation of medial central amygdala neurons. J. Physiol. 599, 3101–3119. 33871877 10.1113/JP281260 PMC8207704

[55] Manning M, Misicka A, Olma A, Bankowski K, Stoev S, Chini B, Durroux T, Mouillac B, Corbani M, and Guillon G (2012). Oxytocin and vasopressin agonists and antagonists as research tools and potential therapeutics. J. Neuroendocrinol. 24, 609–628. 22375852 10.1111/j.1365-2826.2012.02303.x PMC3490377

[56] Song Z, Borland JM, Larkin TE, O’Malley M, and Albers HE (2016). Activation of oxytocin receptors, but not arginine-vasopressin V1a receptors, in the ventral tegmental area of male Syrian hamsters is essential for the reward-like properties of social interactions. Psychoneuroendocrinology 74, 164–172. 27632574 10.1016/j.psyneuen.2016.09.001 PMC6417503

[57] Waltenspühl Y, Ehrenmann J, Vacca S, Thom C, Medalia O, and Plückthun A (2022). Structural basis for the activation and ligand recognition of the human oxytocin receptor. Nat. Commun. 13, 4153. 35851571 10.1038/s41467-022-31325-0 PMC9293896

[58] Gimpl G, and Fahrenholz F (2001). The oxytocin receptor system: structure, function, and regulation. Physiol. Rev. 81, 629–683. 11274341 10.1152/physrev.2001.81.2.629

[59] Sala M, Braida D, Lentini D, Busnelli M, Bulgheroni E, Capurro V, Finardi A, Donzelli A, Pattini L, Rubino T, (2011). Pharmacologic rescue of impaired cognitive flexibility, social deficits, increased aggression, and seizure susceptibility in oxytocin receptor null mice: a neurobehavioral model of autism. Biol. Psychiatry 69, 875–882. 21306704 10.1016/j.biopsych.2010.12.022

[60] Neumann ID, and Landgraf R (2012). Balance of brain oxytocin and vasopressin: implications for anxiety, depression, and social behaviors. Trends Neurosci. 35, 649–659. 22974560 10.1016/j.tins.2012.08.004

[61] Chini B, Mouillac B, Ala Y, Balestre MN, Trumpp-Kallmeyer S, Hoflack J, Elands J, Hibert M, Manning M, and Jard S (1995). Tyr115 is the key residue for determining agonist selectivity in the V1a vasopressin receptor. EMBO J. 14, 2176–2182. 7774575 10.1002/j.1460-2075.1995.tb07211.x PMC398323

[62] Song Z, and Albers HE (2018). Cross-talk among oxytocin and arginine-vasopressin receptors: Relevance for basic and clinical studies of the brain and periphery. Front. Neuroendocrinol. 51, 14–24. 29054552 10.1016/j.yfrne.2017.10.004 PMC5906207

[63] Lowbridge J, Manning M, Haldar J, and Sawyer WH (1977). Synthesis and some pharmacological properties of [4-threonine, 7-glycine] oxytocin, [1-(L-2-hydroxy-3-mercaptopropanoic acid), 4-threonine, 7-glycine]oxytocin (hydroxy[Thr4, Gly7]oxytocin), and [7-Glycine] oxytocin, peptides with high oxytocic-antidiuretic selectivity. J. Med. Chem. 20, 120–123. 833810 10.1021/jm00211a025

[64] Busnelli M, Bulgheroni E, Manning M, Kleinau G, and Chini B (2013). Selective and potent agonists and antagonists for investigating the role of mouse oxytocin receptors. J. Pharmacol. Exp. Therapeut. 346, 318–327. 10.1124/jpet.113.202994 PMC3716315 23723434

[65] Gillard ER, Coburn CG, de Leon A, Snissarenko EP, Bauce LG, Pittman QJ, Hou B, and Currás-Collazo MC (2007). Vasopressin autoreceptors and nitric oxide-dependent glutamate release are required for somatodendritic vasopressin release from rat magnocellular neuroendocrine cells responding to osmotic stimuli. Endocrinology 148, 479–489. 17082256 10.1210/en.2006-0995

[66] Goodson JL, and Thompson RR (2010). Nonapeptide mechanisms of social cognition, behavior and species-specific social systems. Curr. Opin. Neurobiol. 20, 784–794. 20850965 10.1016/j.conb.2010.08.020

[67] Lukas M, and Neumann ID (2013). Oxytocin and vasopressin in rodent behaviors related to social dysfunctions in autism spectrum disorders. Behav. Brain Res. 251, 85–94. 22981649 10.1016/j.bbr.2012.08.011

[68] Grundwald NJ, Benítez DP, and Brunton PJ (2016). Sex-Dependent Effects of Prenatal Stress on Social Memory in Rats: A Role for Differential Expression of Central Vasopressin-1a Receptors. J. Neuroendocrinol. 28. 10.1111/jne.12343 PMC4950027 26613552

[69] Nair HP, Gutman AR, Davis M, and Young LJ (2005). Central oxytocin, vasopressin, and corticotropin-releasing factor receptor densities in the basal forebrain predict isolation potentiated startle in rats. J. Neurosci. 25, 11479–11488. 16339041 10.1523/JNEUROSCI.2524-05.2005 PMC6725901

[70] Daniel SE, and Rainnie DG (2016). Stress Modulation of Opposing Circuits in the Bed Nucleus of the Stria Terminalis. Neuropsychopharmacology 41, 103–125. 26096838 10.1038/npp.2015.178 PMC4677121

[71] Wahis J, Baudon A, Althammer F, Kerspern D, Goyon S, Hagiwara D, Lefevre A, Barteczko L, Boury-Jamot B, Bellanger B, (2021). Astrocytes mediate the effect of oxytocin in the central amygdala on neuronal activity and affective states in rodents. Nat. Neurosci. 24, 529–541. 33589833 10.1038/s41593-021-00800-0

[72] Hasan MT, Althammer F, Silva da Gouveia M, Goyon S, Eliava M, Lefevre A, Kerspern D, Schimmer J, Raftogianni A, Wahis J, (2019). A Fear Memory Engram and Its Plasticity in the Hypothalamic Oxytocin System. Neuron 103, 133–146.e8. 31104950 10.1016/j.neuron.2019.04.029

[73] Rasie Abdullahi P, Eskandarian S, Ghanbari A, and Rashidy-Pour A (2018). Oxytocin receptor antagonist atosiban impairs consolidation, but not reconsolidation of contextual fear memory in rats. Brain Res. 1695, 31–36. 29802839 10.1016/j.brainres.2018.05.034

[74] Fam J, Holmes N, Delaney A, Crane J, and Westbrook RF (2018). Oxytocin receptor activation in the basolateral complex of the amygdala enhances discrimination between discrete cues and promotes configural processing of cues. Psychoneuroendocrinology 96, 84–92. 29909294 10.1016/j.psyneuen.2018.06.006

[75] Missig G, Ayers LW, Schulkin J, and Rosen JB (2010). Oxytocin Reduces Background Anxiety in a Fear-Potentiated Startle Paradigm. Neuropsychopharmacology 35, 2607–2616. 20844476 10.1038/npp.2010.155 PMC3055566

[76] Ayers LW, Missig G, Schulkin J, and Rosen JB (2011). Oxytocin Reduces Background Anxiety in a Fear-Potentiated Startle Paradigm: Peripheral vs Central Administration. Neuropsychopharmacology 36, 2488–2497. 21796104 10.1038/npp.2011.138 PMC3194076

[77] Ayers L, Agostini A, Schulkin J, and Rosen JB (2016). Effects of oxytocin on background anxiety in rats with high or low baseline startle. Psychopharmacology 233, 2165–2172. 27004789 10.1007/s00213-016-4267-0 PMC4864502

[78] Neumann ID, and Slattery DA (2016). Oxytocin in General Anxiety and Social Fear: A Translational Approach. Biol. Psychiatry 79, 213–221. 26208744 10.1016/j.biopsych.2015.06.004

[79] Walker DL, and Davis M (2008). Role of the extended amygdala in short-duration versus sustained fear: a tribute to Dr. Lennart Heimer. Brain Struct. Funct. 213, 29–42. 18528706 10.1007/s00429-008-0183-3

[80] Davis M, Walker DL, Miles L, and Grillon C (2010). Phasic vs Sustained Fear in Rats and Humans: Role of the Extended Amygdala in Fear vs Anxiety. Neuropsychopharmacology 35, 105–135. 19693004 10.1038/npp.2009.109 PMC2795099

[81] Kim S-Y, Adhikari A, Lee SY, Marshel JH, Kim CK, Mallory CS, Lo M, Pak S, Mattis J, Lim BK, (2013). Diverging neural pathways assemble a behavioural state from separable features in anxiety. Nature 496, 219–223. 23515158 10.1038/nature12018 PMC6690364

[82] Yamauchi N, Takahashi D, Sugimura YK, Kato F, Amano T, and Minami M (2018). Activation of the neural pathway from the dorsolateral bed nucleus of the stria terminalis to the central amygdala induces anxiety-like behaviors. Eur. J. Neurosci. 48, 3052–3061. 30240530 10.1111/ejn.14165

[83] Fodor A, Kovács KB, Balázsfi D, Klausz B, Pintér O, Demeter K, Daviu N, Rabasa C, Rotllant D, Nadal R, and Zelena D (2016). Depressive- and anxiety-like behaviors and stress-related neuronal activation in vasopressin-deficient female Brattleboro rats. Physiol. Behav. 158, 100–111. 26939727 10.1016/j.physbeh.2016.02.041

[84] Stoehr JD, Cheng SW, and North WG (1993). Homozygous Brattleboro rats display attenuated conditioned freezing responses. Neurosci. Lett. 153, 103–106. 8510816 10.1016/0304-3940(93)90087-2

[85] Ambrogi Lorenzini C, Bucherelli C, Giachetti A, and Tassoni G (1988). Aversive conditioning of homozygous and heterozygous D.I. Brattleboro rats in the light-dark box. Physiol. Behav. 42, 439–445. 3393603 10.1016/0031-9384(88)90173-4

[86] Pitkow LJ, Sharer CA, Ren X, Insel TR, Terwilliger EF, and Young LJ (2001). Facilitation of affiliation and pair-bond formation by vasopressin receptor gene transfer into the ventral forebrain of a monogamous vole. J. Neurosci. 21, 7392–7396. 11549749 10.1523/JNEUROSCI.21-18-07392.2001 PMC6762997

[87] Bielsky IF, Hu S-B, Szegda KL, Westphal H, and Young LJ (2004). Profound impairment in social recognition and reduction in anxiety-like behavior in vasopressin V1a receptor knockout mice. Neuropsychopharmacology 29, 483–493. 14647484 10.1038/sj.npp.1300360

[88] Egashira N, Tanoue A, Matsuda T, Koushi E, Harada S, Takano Y, Tsujimoto G, Mishima K, Iwasaki K, and Fujiwara M (2007). Impaired social interaction and reduced anxiety-related behavior in vasopressin V1a receptor knockout mice. Behav. Brain Res. 178, 123–127. 17227684 10.1016/j.bbr.2006.12.009

[89] Appenrodt E, Schnabel R, and Schwarzberg H (1998). Vasopressin administration modulates anxiety-related behavior in rats. Physiol. Behav. 64, 543–547. 9761230 10.1016/s0031-9384(98)00119-x

[90] Manning M, Misicka A, Olma A, Bankowski K, Stoev S, Chini B, Durroux T, Mouillac B, Corbani M, and Guillon G (2012). Oxytocin and Vasopressin Agonists and Antagonists as Research Tools and Potential Therapeutics: Oxytocin and vasopressin agonists and antagonists. J. Neuroendocrinol. 24, 609–628. 22375852 10.1111/j.1365-2826.2012.02303.x PMC3490377

[91] Whylings J, Rigney N, de Vries GJ, and Petrulis A (2021). Reduction in vasopressin cells in the suprachiasmatic nucleus in mice increases anxiety and alters fluid intake. Horm. Behav. 133, 104997. 34062279 10.1016/j.yhbeh.2021.104997 PMC8529700

[92] Buijs RM, Hurtado-Alvarado G, and Soto-Tinoco E (2021). Vasopressin: An output signal from the suprachiasmatic nucleus to prepare physiology and behaviour for the resting phase. J. Neuroendocrinol. 33, e12998. 34189788 10.1111/jne.12998

[93] Gillette MU, and Reppert SM (1987). The hypothalamic suprachiasmatic nuclei: circadian patterns of vasopressin secretion and neuronal activity in vitro. Brain Res. Bull. 19, 135–139. 3651837 10.1016/0361-9230(87)90176-6

[94] Gizowski C, Zaelzer C, and Bourque CW (2016). Clock-driven vasopressin neurotransmission mediates anticipatory thirst prior to sleep. Nature 537, 685–688. 27680940 10.1038/nature19756

[95] Bredewold R, and Veenema AH (2018). Sex differences in the regulation of social and anxiety-related behaviors: insights from vasopressin and oxytocin brain systems. Curr. Opin. Neurobiol. 49, 132–140. 29518698 10.1016/j.conb.2018.02.011 PMC6055524

[96] Whylings J, Rigney N, Peters NV, de Vries GJ, and Petrulis A (2020). Sexually dimorphic role of BNST vasopressin cells in sickness and social behavior in male and female mice. Brain Behav. Immun. 83, 68–77. 31550501 10.1016/j.bbi.2019.09.015 PMC6906230

[97] Murillo DG, Zhao Y, Rogovin OS, Zhang K, Hu AW, Kim MR, Chen S, Wang Z, Keeley ZC, Shin DI, (2022). A Platform-Independent Data Interface and Database for Neuroscience Physiology and Imaging Experiments. eNeuro 24, ENEURO.0073–21.2022. 10.1523/ENEURO.0073-21.2022 PMC8874953 35074827

[98] Zhang L, Zetter MA, Hernández VS, Hernández-Pérez OR, Jáuregui-Huerta F, Krabichler Q, and Grinevich V (2024). Morphological Signatures of Neurogenesis and Neuronal Migration in Hypothalamic Vasopressinergic Magnocellular Nuclei of the Adult Rat. Int. J. Mol. Sci. 25, 6988. 39000096 10.3390/ijms25136988 PMC11241681

[99] Pomrenze MB, Millan EZ, Hopf FW, Keiflin R, Maiya R, Blasio A, Dadgar J, Kharazia V, De Guglielmo G, Crawford E, (2015). A Transgenic Rat for Investigating the Anatomy and Function of Corticotrophin Releasing Factor Circuits. Front. Neurosci. 9, 487. 26733798 10.3389/fnins.2015.00487 PMC4689854

[100] Pomrenze MB, Tovar-Diaz J, Blasio A, Maiya R, Giovanetti SM, Lei K, Morikawa H, Hopf FW, and Messing RO (2019). A Corticotropin Releasing Factor Network in the Extended Amygdala for Anxiety. J. Neurosci. 39, 1030–1043. 30530860 10.1523/JNEUROSCI.2143-18.2018 PMC6363927

[101] Griffante C, Green A, Curcuruto O, Haslam CP, Dickinson BA, and Arban R (2005). Selectivity of d[Cha4]AVP and SSR149415 at human vasopressin and oxytocin receptors: evidence that SSR149415 is a mixed vasopressin V1b/oxytocin receptor antagonist. Br. J. Pharmacol. 146, 744–751. 16158071 10.1038/sj.bjp.0706383 PMC1751202

[102] Desai NS, Siegel JJ, Taylor W, Chitwood RA, and Johnston D (2015). MATLAB-based automated patch-clamp system for awake behaving mice. J. Neurophysiol. 114, 1331–1345. 26084901 10.1152/jn.00025.2015 PMC4725114

[103] Dabrowska J, Martinon D, Moaddab M, and Rainnie DG (2016). Targeting Corticotropin-Releasing Factor Projections from the Oval Nucleus of the Bed Nucleus of the Stria Terminalis Using Cell-Type Specific Neuronal Tracing Studies in Mouse and Rat Brain. J. Neuroendocrinol. 28. 10.1111/jne.12442 PMC5362295 27805752

[104] Krashes MJ, Koda S, Ye C, Rogan SC, Adams AC, Cusher DS, Maratos-Flier E, Roth BL, and Lowell BB (2011). Rapid, reversible activation of AgRP neurons drives feeding behavior in mice. J. Clin. Investig. 121, 1424–1428. 21364278 10.1172/JCI46229 PMC3069789

[105] Paxinos G, and Watson C (2009). The Rat Brain in Stereotaxic Coordinates (Amsterdam: Elsevier, Academic Press).

[106] Yang P, Wang Z, Zhang Z, Liu D, Manolios EN, Chen C, Yan X, Zuo W, and Chen N (2018). The extended application of The Rat Brain in Stereotaxic Coordinates in rats of various body weight. J. Neurosci. Methods 307, 60–69. 29960030 10.1016/j.jneumeth.2018.06.026

[107] Ben-Barak Y, Russell JT, Whitnall MH, Ozato K, and Gainer H (1985). Neurophysin in the hypothalamo-neurohypophysial system. I. Production and characterization of monoclonal antibodies. J. Neurosci. 5, 81–97. 3880813 10.1523/JNEUROSCI.05-01-00081.1985 PMC6565074

[108] Williford KM, Taylor A, Melchior JR, Yoon HJ, Sale E, Negasi MD, Adank DN, Brown JA, Bedenbaugh MN, Luchsinger JR, (2023). BNST PKCδ neurons are activated by specific aversive conditions to promote anxiety-like behavior. Neuropsychopharmacology 48, 1031–1041. 10.1038/s41386-023-01569-5. 36941364 PMC10209190

[109] Schneider CA, Rasband WS, and Eliceiri KW (2012). NIH Image to ImageJ: 25 years of image analysis. Nat. Methods 9, 671–675. 22930834 10.1038/nmeth.2089 PMC5554542

[110] Zaelzer C, Gizowski C, Salmon CK, Murai KK, and Bourque CW (2018). Detection of activity-dependent vasopressin release from neuronal dendrites and axon terminals using sniffer cells. J. Neurophysiol. 120, 1386–1396. 29975164 10.1152/jn.00467.2017

[111] Thirouin ZS, and Bourque CW (2021). Mechanism and function of phasic firing in vasopressin-releasing magnocellular neurosecretory cells. J. Neuroendocrinol. 33, e13048. 34672042 10.1111/jne.13048

[112] Walker DL, and Davis M (2002). Quantifying fear potentiated startle using absolute versus proportional increase scoring methods: implications for the neurocircuitry of fear and anxiety. Psychopharmacology 164, 318–328. 12424556 10.1007/s00213-002-1213-0

