# Supplementary material for: Vasopressin and oxytocin excite BNST neurons via oxytocin receptors, which reduce anxious arousal
Source: Cell Rep. Author manuscript; Available in PMC 2025 Jul 25. (PMC12294564; doi:10.1016/j.celrep.2025.115768)
Supplement: 1 [file NIHMS2092650-supplement-1.pdf]

Cell Reports, Volume *44*

Supplemental information

Vasopressin and oxytocin excite

BNST neurons via oxytocin receptors,

which reduce anxious arousal

Walter Francesconi, Valentina Olivera-Pasilio, Fulvia Berton, Susan L. Olson, Rachel Chudoba, Lorena M. Monroy, Quirin Krabichler, Valery Grinevich, and Joanna Dabrowska

Supplemental Items

Table S1. *The effect of OTR and V1R activation on intrinsic membrane properties of Type I-III BNST$_{DL}$ neurons*

Type of Neuron	Treatment	Membrane Properties	Pre-Treatment (Mean ± SEM)	Post-Treatment (Mean ± SEM)	Statistical Analysis	p-value	n
**Type I**	**AVP**	RMP (mV)	-59.1±1.77	-53.87±1.25	F(2, 15)=6.340	0.0101	9
		Rin (MΩ)	139.54±11.74	165.42±16.62	F(1.774, 13.31)=5.695	0.0187	9
		Rh (pA)	27.08±3.24	15.42±2.01	F(2, 13)=7.374	0.0072	8
		First-spike Th (mV)	-35.83±0.79	-35.80±0.65	F(1.300, 9.750)=0.6786	0.4681	9
		First-spike Lat (ms)	221.50±56.41	137.50±33.78	F(2, 15)=7.393	0.0058	9
OTR antagonist	**OTA + AVP**	RMP (mV)	-63.17±1.48	-57.04±2.15	F(1.088, 9.788)=6.851	0.0244	10
		Rin (MΩ)	191.60±15.27	202.60±14.01	F(1.719, 15.47)=5.789	0.0162	10
		Rh (pA)	36.58±4.16	30.17±4.73	F(1.584, 14.26)=1.938	0.1840	10
		First-spike Th (mV)	-35.38± 0.71	-35.72±0.88	F(1.720, 15.48)=0.3976	0.6484	10
		First-spike Lat (ms)	188.20±33.61	134.30±32.14	F(1.967, 17.70)=4.366	0.0293	9
V1aR antagonist	**SR49059 + AVP**	RMP (mV)	-64.22 ± 1.93	-62.47 ± 2.12	F(1.571, 12.57)=5.548	0.0240	9
		Rh (pA)	45.37 ± 7.00	38.61 ± 6.76	F(1.806, 14.45)=6.361	0.0120	9
		Rin (MΩ)	180.1 ± 15.50	202.1 ± 16.27	F(1.216, 9.730)=3.353	0.0930	9
		First-spike Lat (ms)	161.1 ± 18.26	143.4 ± 21.08	F(1.273, 10.19)=0.7476	0.4393	9
		Th (mV)	-36.23 ± 1.51	-34.88 ± 1.72	F(1.260, 10.08)=2.523	0.1401	9
V1bR antagonist	**Nelivaptan + AVP**	RMP (mV)	-63.13±2.28	-56.76±2.73	F(1.352, 12.17)=15.50	0.0010	10
		Rin (MΩ)	189.5±16.30	214.6±17.92	F(1.421, 12.79)=4.299	0.0480	10
		Rh (pA)	39.50±5.70	31.00±5.36	F(1.387, 12.48)=8.143	0.0093	10
		First-spike Th (mV)	-30.28±1.69	-28.59±2.22	F(1.292, 11.63)=2.541	0.1335	10
		First-spike Lat (ms)	187.2±28.40	113.6±18.15	F(1.707, 15.37)=6.963	0.0090	10
**Type II**	**AVP**	RMP (mV)	-53.12±1.46	-51.29±1.69	F(1.155, 6.927)=2.219	0.1815	8
		Rin (MΩ)	135.9±15.83	151.3±21.57	F(2,12)= 3.348	0.0699	8
		Rh (pA)	25.10±5.39	25.62±8.73	F(1.016, 6.095)=0.08300	0.7867	8

Type of Neuron	Treatment	Membrane Properties	Pre-Treatment (Mean ± SEM)	Post-Treatment (Mean ± SEM)	Statistical Analysis	p-value	n
		First-spike Th (mV)	-36.82±0.82	-34.65±1.53	F(1.818, 10.91)=1.199	0.3336	8
		First-spike Lat (ms)	97.3±7.3	75.27±8.7	F(1.403, 8.415)=1.146	0.3397	8
**Type III**	**AVP**	RMP (mV)	-68.34±1.36	-65±1.93	F(2, 15)=5.717	0.0143	10
		Rin (MΩ)	108.8±10.29	128.9±13.83	F(1.602, 5.608)=30.90	0.0011	5
		Rh (pA)	75.15±9.45	63.5±9.65	F(1.114, 8.356)=12.18	0.0068	10
		First-spike Th (mV)	-33.12±1.89	-33.46±1.92	F(1.187, 8.902)=0.4559	0.5495	10
		First-spike Lat (ms)	419.08±64.09	257.5±52.13	F(1.869, 13.08)=11.79	0.0014	10
	**OTR/V1aR Antagonist + AVP**	RMP (mV)	-69.79±1.21	-67.97±1.17	F(1.211, 7.264)=3.616	0.0936	7
		Rin (MΩ)	94.74±16.92	97.37±17.56	F(1.904, 11.42)=2.037	0.1761	7
		Rh (pA)	88.33±8.97	83.33±4.42	F(1.335, 8.007)=0.8376	0.4213	7
		First-spike Th (mV)	-33.41±0.95	-34.69±0.79	F(1.344, 8.063)=2.478	0.1517	7
		First-spike Lat (ms)	203.2±29.08	176.2±32.81	F(1.174, 7.041)=0.3909	0.5843	7
	**OTA + AVP**	RMP (mV)	-67.37±1.72	-66.59±1.9	F(1.039, 6.235)=0.6159	0.4675	7
		Rin (MΩ)	110.99±8.5	132.79±15.	F(1.042, 4.169)=5.504	0.0758	5
		Rh (pA)	77.02±10.75	75.71±12.74	F(1.197, 7.185)=0.0635	0.8495	7
		First-spike Th (mV)	-38.86±1.96	-37.58±2.46	F(1.926, 11.56)=1.482	0.2667	7
		First-spike Lat (ms)	327.6±60.45	300.3±60.50	F(2, 10)=0.5830	0.5761	6
	**SR49059 + AVP**	RMP (mV)	-66.95 ± 1.88	-63.94 ± 2.38	F(1.341, 9.386)=11.51	0.0052	8
		Rin (MΩ)	159 ± 13.93	181.5 ± 14.85	F(1.764, 12.35)=5.944	0.0179	8
		Rh (pA)	68.96± 7.48	56.67 ± 7.13	F(1.249, 8.745)=7.536	0.0193	8
		First-spike Th (mV)	-34.09 ± 1.33	-33.25 ± 2.33	F(1.844, 12.91)=1.825	0.2014	8
		First-spike Lat (ms)	404 ± 72.91	261.2 ± 65.96	F(1.546, 10.83)=7.551	0.0121	8
	**Nelivaptan + AVP**	RMP (mV)	-74.24 ± 1.46	-69.45 ± 1.74	F(1.331 10.65) = 15.67	0.0014	9
		Rin (MΩ)	124.9 ± 9.76	186.6 ± 24.8	F(1.196, 9.565) = 9.060	0.0114	9

Type of Neuron	Treatment	Membrane Properties	Pre-Treatment (Mean ± SEM)	Post-Treatment (Mean ± SEM)	Statistical Analysis	p-value	n
		Rh (pA)	94.44 ± 5.86	84.26 ± 6.5	F(1.507, 12.06) = 5.441	0.0272	9
		First-spike Th (mV)	-29.9 ± 1.76	-24.24 ± 3.12	F(1.077, 8.613) = 4.558	0.0610	9
		First-spike Lat (ms)	377.2 ± 64.25	248.4 ± 37.86	F(1.539, 12.31) = 4.491	0.0402	9
**Type III**	**OT**	RMP (mV)	-68.54±1.03	-67.79±0.87	F(2, 13)=1.374	0.2875	8
		Rin (MΩ)	137.15±23.61	142.41±26.16	F(0.8062, 3.628)=14.14	0.0251	6
		Rh (pA)	83.02±4.81	69.33±4.74	F(2, 13)=13.22	0.0007	8
		First-spike Th (mV)	-29.27±1.86	-29.49±1.98	F(0.04181, 0.2717)=2.479	0.1480	8
		First-spike Lat (ms)	497.8±63.95	281.05±43.37	F(1.827, 10.96)=11.13	0.0027	8
OTR agonist	**TGOT**	RMP (mV)	-71.86±1.00	-70.00±1.22	F(1.168, 8.177)=5.241	0.0469	9
		Rin (MΩ)	85.79±6.63	91.24±7.02	F(1.076, 4.841)=8.456	0.0338	7
		Rh (pA)	93.61±9.88	82.69±10.97	F(1.776, 11.54)=6.129	0.0174	9
		First-spike Th (mV)	-30.84±0.98	-30.79±1.13	F(1.120, 7.838)=0.6998	0.4439	9
		First-spike Lat (ms)	594.03±76.46	355.24±68.69	F(0.9019, 5.412)=7.726	0.0373	9
V1aR agonist	**FE 201874**	RMP (mV)	-70.72±0.9	-69.44±1.25	F(1.242, 9.312)=3.093	0.1068	10
		Rin (MΩ)	118.21±1.25	117.57±9.69	F(1.219, 6.094)=0.2128	0.70748	7
		Rh (pA)	77.83±9.55	73.66±10.5	F(1.209, 9.066)=1.728	0.2256	10
		First-spike Th (mV)	-30.45±1.22	-31.93±1.1	F(1.550, 11.63)=2.515	0.1312	10
		First-spike Lat (ms)	432.8±65.72	345.3±57.68	F(1.710, 11.12)=3.780	0.0610	9
V1bR agonist	**d[Cha4]-AVP**	RMP (mV)	-70.42±0.84	-69.81±1.15	F(1.987, 12.91)=1.327	0.2988	8
		Rin (MΩ)	105.18±7.85	110.98±10.05	F(0.9314, 6.054)=2.663	0.1532	8
		Rh (pA)	86.25±5.96	81.46±6.2	F(1.160, 7.540)=1.193	0.3195	8
		First-spike Th (mV)	-33.38±1.67	-34.00±1.79	F(0.9257, 6.017)=2.467	0.1666	8
		First-spike Lat (ms)	475.1±59.48	426.6±60.20	F(1.063, 5.848)=10.24	0.0185	8

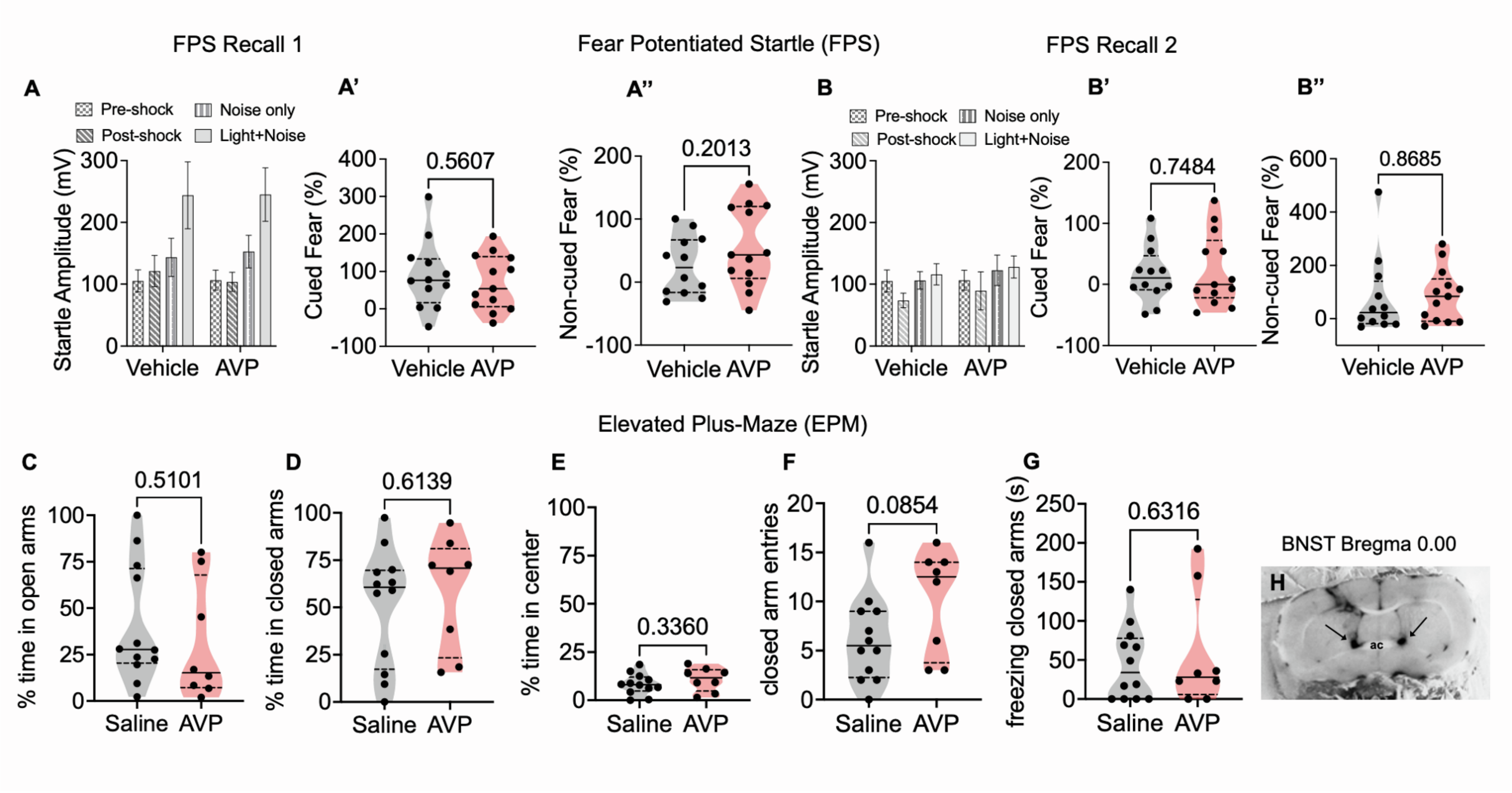
FPS Recall 1
Fear Potentiated Startle (FPS)
FPS Recall 2
A
Pre-shock
Noise only
Post-shock
Light+Noise
Startle Amplitude (mV)
Vehicle
AVP
A'
Cued Fear (%)
0.5607
Vehicle AVP
A''
Non-cued Fear (%)
0.2013
Vehicle AVP
B
Pre-shock
Noise only
Post-shock
Light+Noise
Startle Amplitude (mV)
Vehicle
AVP
B'
Cued Fear (%)
0.7484
Vehicle AVP
B''
Non-cued Fear (%)
0.8685
Vehicle AVP
Elevated Plus-Maze (EPM)
C
% time in open arms
0.5101
Saline AVP
D
% time in closed arms
0.6139
Saline AVP
E
% time in center
0.3360
Saline AVP
F
closed arm entries
0.0854
Saline AVP
G
freezing closed arms (s)
0.6316
Saline AVP
BNST Bregma 0.00
H
ac

Supplemental figure 1 (Fig. S1): AVP infusion into the $BNST_{DL}$ does not affect fear or anxiety-like behaviors.

A-B'': *AVP infusion in the BNST does not affect cued fear or anxious arousal in the FPS*. **A-A''**: During the first FPS recall, there was a significant trial effect of noise-only vs. light+noise (*P*=0.0029, F(1, 23)=11.08, two-way RM ANOVA), but no treatment effects of AVP (n=13) vs. vehicle (n=12) (*P*=0.9140, F(1, 23)=0.01192), nor interaction (*P*=0.8907, F(1, 23)=0.01930). There was also a significant trial effect between the post-shock and noise-only (*P*=0.0085, F(1, 23)=8.281), indicating a non-cued fear expression, but no treatment effect (*P*=0.9029, F(1, 23)=0.01521), nor interaction (*P*=0.2917, F(1, 23)=1.165)**.** No significant differences were found in the percentage change of cued fear (**A**') or non-cued fear (**A''**), or contextual fear (*P*=0.5373, **not shown**) between the AVP vs. vehicle groups. **B-B'':** During the second FPS recall, there was no more significant trial effect between the noise-only and light+noise (*P*=0.5033, F(1, 23)=0.4623), no treatment effect (*P*=0.5721, F(1, 23)=0.3286), and no interaction (*P*=0.8533, F(1, 23)=0.03499). There was a significant trial effect between the post-shock and noise-only (*P*=0.0024, F(1, 23) =9.299), but no treatment effect (*P*=0.5982, F(1, 23)=0.2856), or interaction (*P*=0.9745, F(1, 23)=0.001042). During the second contextual fear recall test in context A, two-way RM ANOVA showed a trial effect between the pre-shock and post-shock (*P*=0.0006, F(1, 23)=16.01), but no treatment effect (*P*=0.7887, F(1, 23)=0.07353), and no interaction (*P*=0.8120, F(1, 23)=0.05786), **not shown**). There were no significant differences in the percentage change of cued fear (**B'**), non-cued fear (**B''**), or contextual fear (*P*=0.7073, **not shown**) between the groups. During the third FPS recall in context B, there was no more significant trial effect between the noise-only and light+noise (*P*=0.0536, F(1, 23)=4.140), but there was a significant trial effect between the post-shock and noise-only (*P*=0.0005, F(1, 23)=16.13), but no treatment effect (*P*=0.8197, F(1, 23)=0.05318), and no interaction (*P*=0.6405, F (1, 23)=0.2240). There were no differences between the percentage change of cued (*P*=0.6509) or non-cued fear (*P*=0.7112) between the groups, **not shown**. During fear conditioning, there were no significant difference in the reactivity to foot-shocks between vehicle- and AVP-injected rats (*P*=0.3836, **not shown**). ***C-G:*** *AVP infusion in the BNST does not affect anxiety-like behavior in the EPM.* After the FPS, the same rats were tested in the EPM after vehicle vs. AVP infusion. There was no difference in time spent in the EPM open-arms (**C**) between rats treated with AVP (n=8) vs. vehicle (n=12), or time spent in the closed-arms (**D**), or time in the center (**E**). No significant differences were found in the number of closed-arms entries (**F**) or time freezing in the closed-arms (**G,** un-paired *t*-test). **H)** Representative section showing proper bilateral cannulas placement in the $BNST_{DL}$, here shown with a Chicago Blue dye injection.

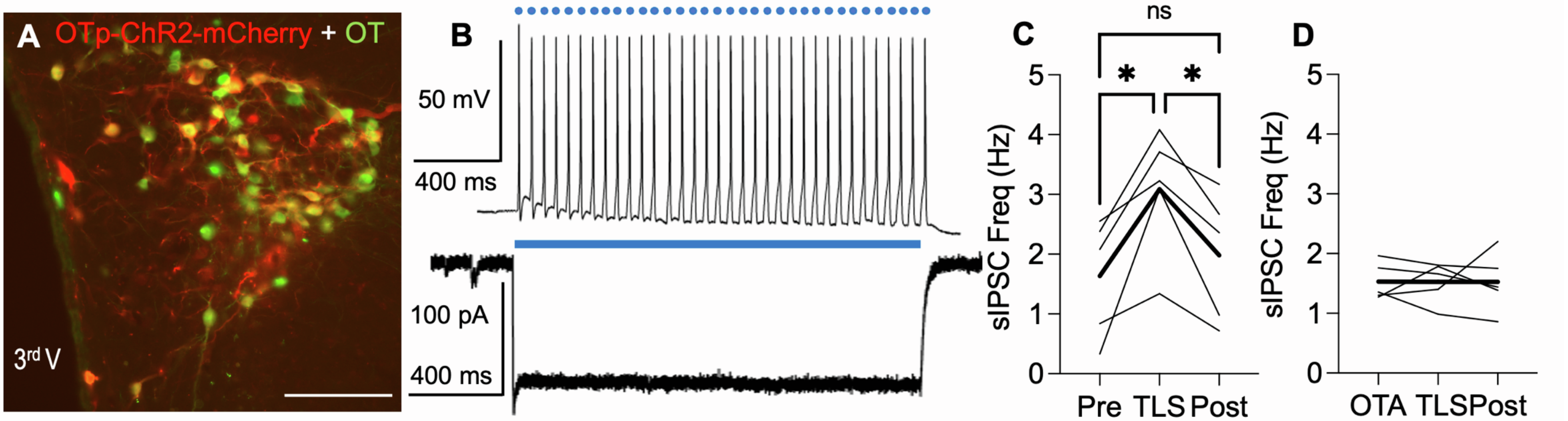

A OTp-ChR2-mCherry + OT
3rd V
B
50 mV
400 ms
100 pA
400 ms
C
ns
*
*
sIPSC Freq (Hz)
0
1
2
3
4
5
Pre TLS Post
D
sIPSC Freq (Hz)
0
1
2
3
4
5
OTA TLS Post

Supplementary figure 2 (Fig. S2): Tetanic light stimulation (TLS) evokes OT release and increases inhibitory synaptic transmission in Type II $BNST_{DL}$ neurons in an OTR-dependent manner.

A: High somatodendritic expression of ChR2-mCherry (red) is observed in hypothalamic OT neurons (mouse anti-OT MAB5296 antibody, green) 3 weeks after *AAV-OTp-ChR2-mCherry* was injected to the PVN in male rats (20x, scale bar 100 µm). **B**: Action potentials recorded from PVN neurons expressing ChR2-mCherry, evoked by 10 ms repeated (30 Hz, 1 sec) blue light stimulation (upper panel) and current induced by 1 sec continuous blue light (bottom panel). **C**: In the $BNST_{DL}$ from rats injected as above, TLS (30 Hz train, single pulse duration 10 ms for 20 sec) increased frequency of spontaneous inhibitory postsynaptic currents (sIPSCs) in Type II $BNST_{DL}$ neurons ($F(1.436, 7.182)=16.86$, $P=0.0028$, one-way ANOVA, n=5) but did not affect sIPSCs amplitude ($F(1.851, 7.404)=2.471$, $P=0.1520$, not shown). This effect mimics a well-established cellular effect of exogenous OT in the $BNST_{DL}$, which was shown to increase frequency of sIPSCs specifically in Type II $BNST_{DL}$ neurons [39] (*Francesconi et al., 2021*). **D**: Notably, TLS did not affect sIPSCs frequency in the presence of selective OTR antagonist, OTA ($F(1.565, 7.823)=0.0003$, $P=0.9985$, n=5). Fine lines represent individual Type II neurons responses, black thick lines - average responses, *$P<0.05$, 3^{rd}V-Third ventricle.

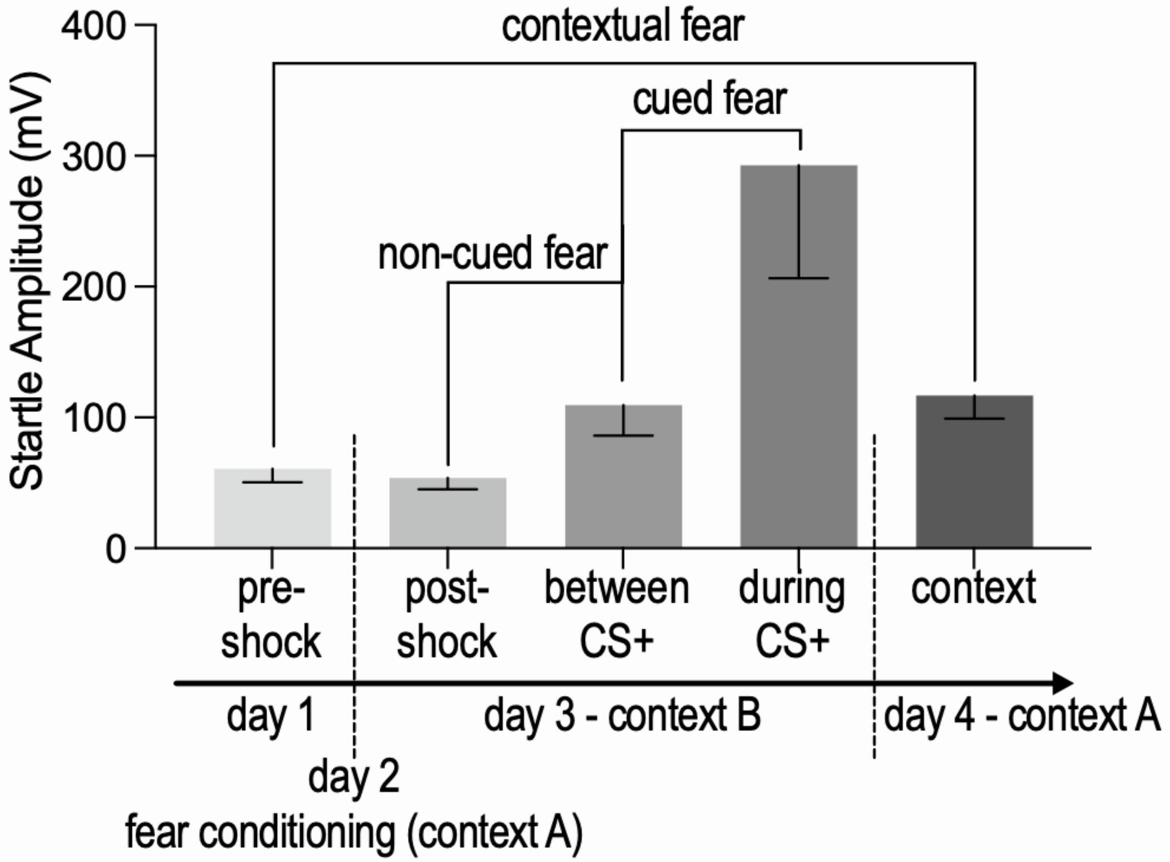
400
300
200
100
0
Startle Amplitude (mV)
contextual fear
cued fear
non-cued fear
pre-shock
post-shock
between CS+
during CS+
context
day 1
day 3 - context B
day 4 - context A
day 2
fear conditioning (context A)

Supplementary figure 3 (Fig. S3): Fear-potentiated startle (FPS) components.

Rats are tested for baseline acoustic startle response ASR, pre-shock, day 1) before fear conditioning on day 2 (context A). On day 3 fear recall is tested in context B: Cued fear is calculated as a percent change score of ASR amplitude from 'between conditioned stimulus (CS+)' to ASR 'during CS+'. Non-cued fear is calculated as a percent change score from post-shock to 'between CS+'. Note, during the fear recall test, post-shock ASR is measured before the first CS+ presentation. On day 4 rats are tested for contextual fear recall in context A with no CS presentations, calculated as percent change score from pre-shock on day 1 to ASR in context A on day 4. Fear recall in context B is tested a total of three times, while contextual fear recall in context A is tested twice, and these tests occur on an alternating basis.
